# Longitudinal associations between parental mental health and the duration of visual attention and facial expressions during at-home parent–infant interactions: a UK birth cohort study

**DOI:** 10.3389/frcha.2025.1638234

**Published:** 2025-10-27

**Authors:** Ilaria Costantini, Daphne Kounali, Iryna Culpin, Marc H. Bornstein, Rebecca M. Pearson

**Affiliations:** 1 Division of Psychiatry, University College London, London, United Kingdom; 2 Centre for Academic Mental Health, Population Health Sciences, University of Bristol, Bristol, United Kingdom; 3 Oxford Clinical Trials Research Unit, Centre for Statistics in Medicine, Nuffield Department of Orthopaedics, Rheumatology and Musculoskeletal Sciences, University of Oxford, Oxford, United Kingdom; 4 Florence Nightingale Faculty of Nursing, Midwifery and Palliative Care, Department of Child and Family Health, King's College London, London, United States; 5 Eunice Kennedy Shriver National Institute of Child Health and Human Development, Bethesda, MD, United States; 6 Institute for Fiscal Studies, London, United Kingdom; 7 Department of Population Health Sciences, Bristol Medical School, University of Bristol, Bristol, United Kingdom; 8 Faculty of Health and Education, School of Psychology, Manchester Metropolitan University, Manchester, United Kingdom

**Keywords:** ALSPAC, parent-infant interactions, depression, personality difficulties, headcams

## Abstract

**Background:**

Parental mental health difficulties have been associated with variation in parent–infant interactions, including facial expressions and visual attention. Most prior research has relied on clinical samples and structured observational settings, limiting ecological validity and generalisability to population-level variation.

**Aims:**

This study aimed to (i) characterise the duration of facial expressions and visual attention behaviours in parents and infants during naturalistic interactions at home, and (ii) explore associations between parental depressive symptoms and personality difficulties—measured prenatally or preconception—and these observed behaviours.

**Methods:**

Interactions were recorded at home using synchronised head-mounted cameras worn by parents and infants. Facial expressions and gaze behaviours were micro-coded for each dyad member using a validated behavioural coding system. Parental depressive symptoms and personality difficulties were assessed using the Edinburgh Postnatal Depression Scale (EPDS) and the Standardised Assessment of Personality—Abbreviated Scale (SAPAS). Associations were estimated using bivariate two-level models, adjusting for relevant covariates and clustering at the dyad level.

**Findings:**

A total of 142 video observations were obtained from 97 families participating in the Avon Longitudinal Study of Parents and Children (ALSPAC), including 102 unique parent–infant dyads. Of the 142 observations, 74% involved mothers as the primary caregiver. Infants were on average 7 months old, and 66% were first-born. We found suggestive evidence that higher parental depressive symptoms and personality difficulties were associated with shorter durations of expressions such as “mock surprise” and “woe face”, and with longer durations of negative affect. Infants of parents with higher depressive symptoms showed longer smiling and increased visual attention to others in the room, potentially reflecting social referencing.

**Conclusions:**

Wearable cameras offer a feasible and ecologically valid method for observing parent–infant interactions in home settings. Findings suggest that variation in parental mental health is associated with differences in both parental and infant emotional and attentional behaviours. These preliminary results underscore the potential of wearable technology for advancing research on early relational processes.

## Introduction

The quality of early parent-infant interactions has been associated with the development of children's emotional regulation and social functioning (1), which in turn have been linked to long-term mental health outcomes in children (2). Improving parent-infant relationships could therefore have major public health implications, with recent estimates suggesting potential savings of £900 million annually in the UK alone (3). Evidence from randomised controlled trials (RCTs) and meta-analyses suggests that interventions targeting early interactions can improve child emotional and behavioural outcomes (4–6). However, these studies have often reported small effect sizes and short-term benefits (4). A more precise understanding of the salient features of parent–infant interaction, and how these relate to parental mental health, may help refine intervention targets and enhance both efficacy and durability of outcomes.

Visual attention and affective facial expressions are key markers of interaction quality in early caregiving. Face-to-face communication has been examined extensively in mother–infant dyads and, to a lesser extent, father–infant dyads (7–10). Facial expressions indeed convey critical affective signals and are thought to support the development of both self-regulation and co-regulation (8, 9). Visual attention and gaze direction likewise are considered to foster emotional attunement and moment-to-moment regulation of arousal within the dyad (9, 11–13). While most research has focused on the contingency and synchrony of parent–infant behaviours, the total amount of time that parents and infants spend maintaining visual attention (e.g., towards the caregiver or the shared object of interaction) and displaying facial expressions offers a simple, complementary index of the stability of engagement within the dyad.

Parental mental health difficulties are a putative risk factor for the quality of parent–infant interactions and later child outcomes (14–18).

Observational studies show that mothers experiencing depression—both current and past—often display reduced sensitivity, greater disengagement, and more negative or coercive behaviours in interactions with their infants (19–21), and that mothers with personality difficulties, including borderline personality disorder, similarly exhibit reduced sensitivity (22). With respect to visual attention and facial expressions, mothers with depression and/or personality difficulties gaze less at their infants (23), show fewer positive expressions (24–26), and disengage more quickly from infant distress (27). Mothers with elevated depressive symptoms also show reduced facial empathy for infant distress (e.g., fewer “woe faces”), although they spend more time looking directly at their infant's face (28). Infants of depressed mothers likewise exhibit reduced social gaze, more gaze aversion (29), and less smiling (30). Emerging evidence further suggests that socio-affective processing undergoes adaptive reorganisation during pregnancy (31, 32), and that mental health problems at this stage may disrupt such neurocognitive changes, with downstream consequences for bonding and caregiving (31, 33). These findings underscore the need to examine whether difficulties arising during pregnancy—not only postpartum—are associated with later disruptions in parent–infant interactions.

Despite these advances, the existing literature has important limitations. Several studies are based on clinical samples (25, 26)—parents with a diagnosis or receiving treatment—or apply clinical cut-offs to continuous measures (29, 30), limiting generalisability to community populations and reducing statistical power. Observations are also frequently conducted in structured laboratories (24, 26) or in the presence of an observer in the family home (23, 30), contexts that may alter behaviour and increase social desirability. It therefore remains unclear whether similar patterns are evident when parental mental health is measured dimensionally in community samples, and whether naturalistic, at-home recordings without an observer present might reveal different associations.

Naturalistic observation of parent–infant behaviour offers a way to overcome these challenges. Wearable cameras offer an ecologically valid method for unobtrusively capturing spontaneous interactions in the home environment, reducing reactivity and observer bias (34). Data collected in this way may yield a more accurate picture of everyday dyadic behaviours and help identify specific affective and attentional markers associated with parental mental health. Moreover, such first-person footage can be integrated into intervention delivery—for example, through video-feedback interventions adapted for home settings—to enhance caregiver engagement and relevance (35, 36).

In the present study, we used footage collected via head-mounted cameras worn by parents and infants during interactions in the home, within a population-based cohort. Video footage was micro-coded using a structured scheme based on the Mental Health Intergenerational Transmission (MHINT) manual (37) to quantify the duration and type of facial expressions and visual attention in both parents and infants (Objective 1). By examining these behaviours in detail, the study also aimed to explore whether and how parental prenatal or preconception depressive symptoms and personality difficulties are associated with duration of visual attention and facial expression behaviours (Objective 2). We hypothesised that higher parental depressive symptoms and greater personality difficulties would be associated with reduced facial mirroring/empathic responding during parent–infant interactions—operationalised as shorter durations of empathic expressions (e.g., *woe face*) and other concordant, affiliative displays (e.g., *mock surprise* in playful contexts)—and with greater negative affective display, operationalised as longer durations of *negative* facial expressions (e.g., distress/anger/fear) and shorter durations of *smiling/positive* facial expressions. We also hypothesised that infants of parents with higher depressive symptoms and personality difficulties would show less positive affect (shorter *smile/positive* durations) and more avoidance-related behaviours (e.g., reduced looking at caregiver).

## Methods

### Sample

We used data from the Avon Longitudinal Study of Parents and Children (ALSPAC), a multigenerational prospective birth cohort study based in South-West England (38). 14,541 pregnant mothers residing in South-West England with expected dates of delivery between 1 April 1991 and 31 December 1992 were recruited (ALSPAC-G0). The total sample size included 15,454 pregnancies; 14,901 babies were alive at 1 year of age (ALSPAC-G1). Full cohort details are provided in Boyd et al. (39), Fraser et al. (38), and further updates are available in Lawlor et al. (40) and Northstone et al. (41).

In 2012, the recruitment of the second generation of ALSPAC (ALSPAC-G2) started: the aim was to recruit all the children of ALSPAC-G1 as well as to recruit all the (non-ALSPAC) partners of the ASLPAC-G1 parents. Data were collected from both parents (at least one of whom was a G1 participant) and their children. The study website contains details of all data available through a fully searchable data dictionary (http://www.bristol.ac.uk/alspac/researchers/our-data/). Study data were collected and managed using REDCap electronic data capture tools hosted at the University of Bristol (42). REDCap (Research Electronic Data Capture) is a secure, web-based software platform designed to support data capture for research studies. Ethical approval for the study was obtained from the ALSPAC Law and Ethics Committee and the Local Research Ethics Committees and written informed consent was provided. Further details on recruitment are available in Supplementary Appendix, p. 4.

#### Recruitment into the headcams study

Recruitment of mothers into the headcams study began on July 2016. Data collection was halted due to COVID-19 from March 2020 to April 2021, when virtual visits were introduced. From January 2022, face-to-face clinic visits were re-introduced. 439 mothers were invited to participate in the study, of whom 241 (55%) accepted the invitation and 155 (35%) agreed to record their interactions with their infant using the headcams at home. Overall, 283 fathers were invited to attend, with 154 (54%) fathers consenting to participate, and 86 (30%) fathers providing video footage of father-infant interactions.

**Figure 1:**
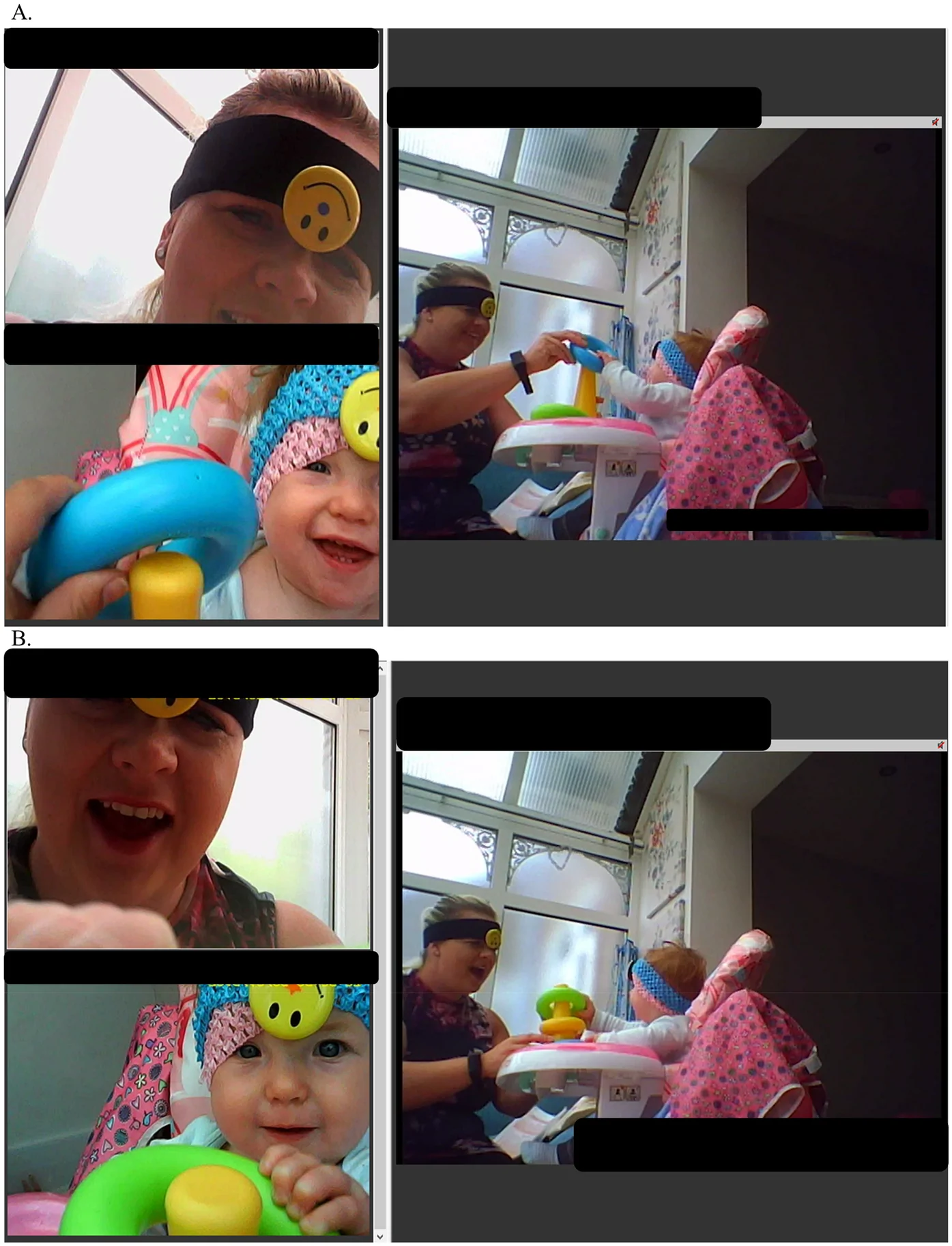
Perspectives obtained using the “old” (pre-COVID-19) wearable cameras. Note. Written informed consent was obtained from the minor's legal guardian for the publication of any potentially identifiable images. **(A)** Depicts the stacking task activity, with perspectives from the infant headcam (top left), parent headcam (bottom left), and a third-person “photo frame” camera (right). At this timepoint, both infant and parent display positive facial expressions, with mutual gaze. **(B)** Shows the same interaction, during which the parent is coded as showing a “mock surprise” expression.

**Figure 2:**
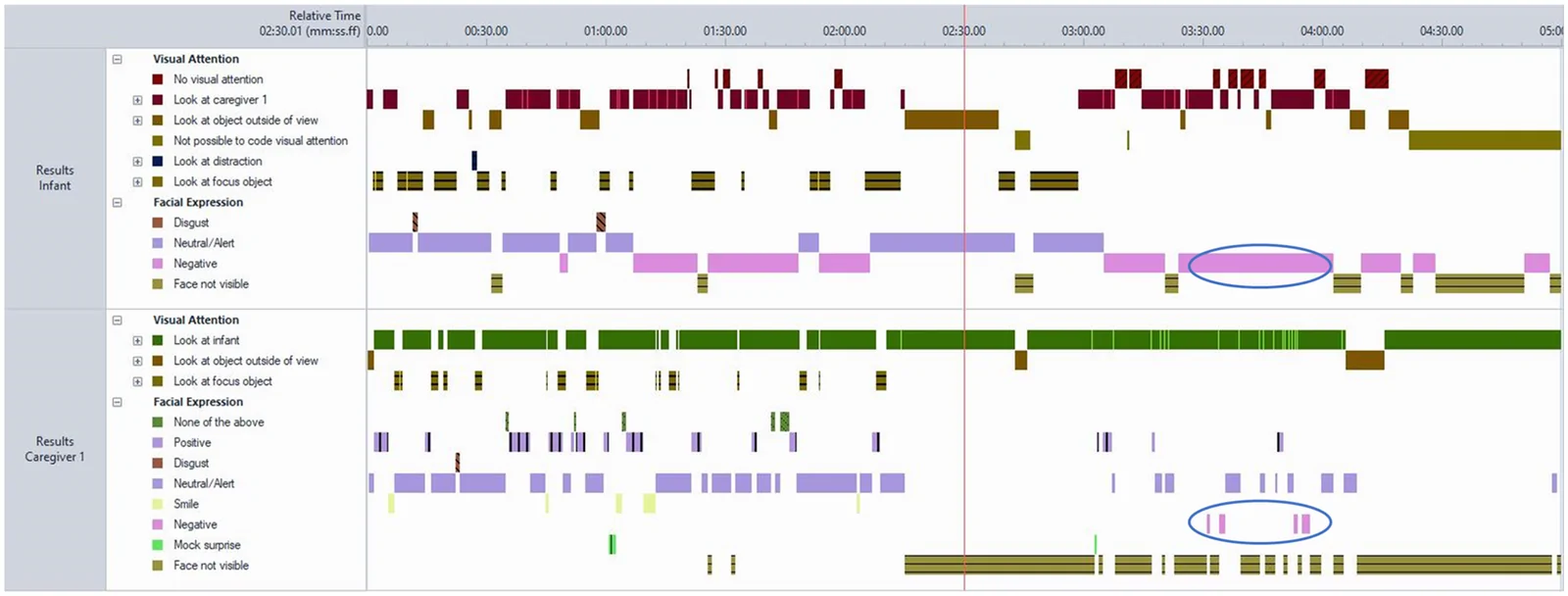
Visualisation of a single observation using sequential timed-event coding. Note. The figure illustrates parallel behavioural streams coded continuously and independently for each subject and behavioural group. Each code is mutually exclusive and exhaustive within its category (e.g., facial expression or visual attention). Circled events highlight co-occurrence of parent and infant negative facial expressions. Total duration of behaviours was computed as a proportion of observable time, adjusting for footage length and any periods where visibility was obstructed.

For the purposes of this study, 97 unique families, which included 102 unique caregiver-infant dyads, were analysed using Noldus Observer XT® 16.0 software (43). Two generations of head—mounted cameras were used—earlier Bogdan DVR devices (720 × 480 px, 60° field of view) and later Ucam247 WearCams (1280 × 720 px, 85° field of view)—both worn by parent and infant to capture simultaneous first—person perspectives of interaction (62). Coding followed a continuous timed event-based approach, where all behaviours were categorised using mutually exclusive and exhaustive codes within each behavioural group (e.g., facial expression, visual attention), allowing parallel coding across domains (44). For example, at any timepoint, an individual was coded for both facial expression and gaze direction (Figure 1). Only one code per behavioural group was assigned at any moment, ensuring precise capture of behavioural states (Figure 2). The outcome variables represented the proportion of time during the interaction in which each behaviour was observed, separately for infants and caregivers.

#### Outcomes

Full operational definitions on the specific behavioural groups for both facial expression and visual attention, individual behaviours, and modifiers are provided in the MHINT manual (37) and the Supplementary Appendix, p 7. Inter-rater reliability was assessed through double-coding of a subset of videos. Agreement between coders was evaluated with Cohen's kappa (*κ*), calculated separately for each behavioural group. Reliability was generally high, with *κ* values ranging from 0.67 to 0.85 for facial expressions to 0.75–0.84 for visual attention in mothers and fathers, and exceeding 0.90 for several other groups, see Table 1 in (45).

##### Facial expressions

Coders categorised infant and parent facial expressions as one of: neutral/alert; smile; positive (non-smile); negative; disgust; surprise; woe face; mock surprise; none of the above; or face not visible. Briefly: *neutral/alert* is used when no clear positive or negative affect is observed (relaxed musculature, open eyes); *smile* indicates a clear, unambiguous Duchenne smile involving both mouth and eyes (activation of zygomaticus and orbicularis oculi); *positive (non-smile)* refers to joy/interest/excitement without a full Duchenne smile (e.g., open-mouth positive attention); *negative* denotes distress-related or aversive states (e.g., sadness, discomfort/pain, anger, fear) typically showing lowered brows, raised cheeks, and a horizontally stretched mouth; *disgust* is coded when nose-wrinkling/upper-lip raise or gape/turn-away typical of revulsion is present and is kept distinct from *negative* to disentangle these states—especially for infants during feeding behaviours; *surprise* is coded when raised brows, raised upper eyelids, and an open mouth signal novelty; *woe face* (parents only) captures empathic concern (slightly downturned mouth corners with pursed lips), typically in response to infant distress; *mock surprise* (parents only) reflects playful, exaggerated surprise not indicating genuine novelty; *none of the above* is used for mixed/atypical expressions (e.g., sneezing, coughing); and *face not visible* is used when facial visibility is insufficient (<1 eye and mouth visible, duration <1 s, or out of frame).

##### Visual attention

Coders classified gaze as: look at infant; look at caregiver 1; look at caregiver 2; look at focus object (task-relevant item, e.g., food, toy, book); look at other object; look at object outside of view (object not visible or uncertain); look at sibling; look at other person; look at distraction (with modifiers such as phone, TV, computer, pet); no visual attention (no codable fixation); or not possible to code. Caregiver 1 is defined as the primary caregiver on camera and in interaction with the infant, while Caregiver 2 indicate the other caregiver present during the interaction (45).

#### Exposures

##### Antenatal or preconception depressive symptoms

Symptoms of maternal and paternal depression were assessed using the Edinburgh Postnatal Depression Scale [EPDS (46)], a 10-item self-reported questionnaire validated for use during the perinatal period (47). Depressive symptoms were primarily measured in pregnancy (97%) (40), with the remaining scores being measured in preconception via annual questionnaires, which consisted of general questions sent out to all participants once a year.

##### Personality difficulties

Personality difficulties were measured using the Standardised Assessment of Personality—Abbreviated Scale (SAPAS) (48). The SAPAS is an 8-item screening tool designed to assess core features of personality disorder, such as impulsivity, mistrust, and emotional instability. Responses are binary (yes/no), with higher scores indicating greater personality difficulties. The SAPAS was collected at the 24 years of age clinic visit as part of the original ALSPAC-G1 data collection. 93% of the parents had SAPAS measured preconception or in pregnancy, and the remaining 7% of parents had SAPAS measured postpartum before, during, or after headcam data were collected.

Continuous scores were used in all analyses with higher scores indicating more severe depressive symptoms and more personality difficulties. In the present study, we use the term “personality difficulties” to refer to enduring patterns of cognition, affect, and behaviour.

#### Covariates

In all models we adjusted for covariates that could confound or explain variance in behavioural outcomes. Parental age was included as a putative confounder given its association with both parental mental health and parent–infant interaction quality. Additional covariates were specified to account for measurement or contextual factors: headcam type (old vs. new), activity type (i.e., feeding, free-play, stacking-task, mixed), child age at the time of the interaction, child sex as reported by the parent at birth, birth order, and who was the primary caregiver present during the interaction (i.e., mother vs. father).

### Statistical analyses

**Figure 3:**
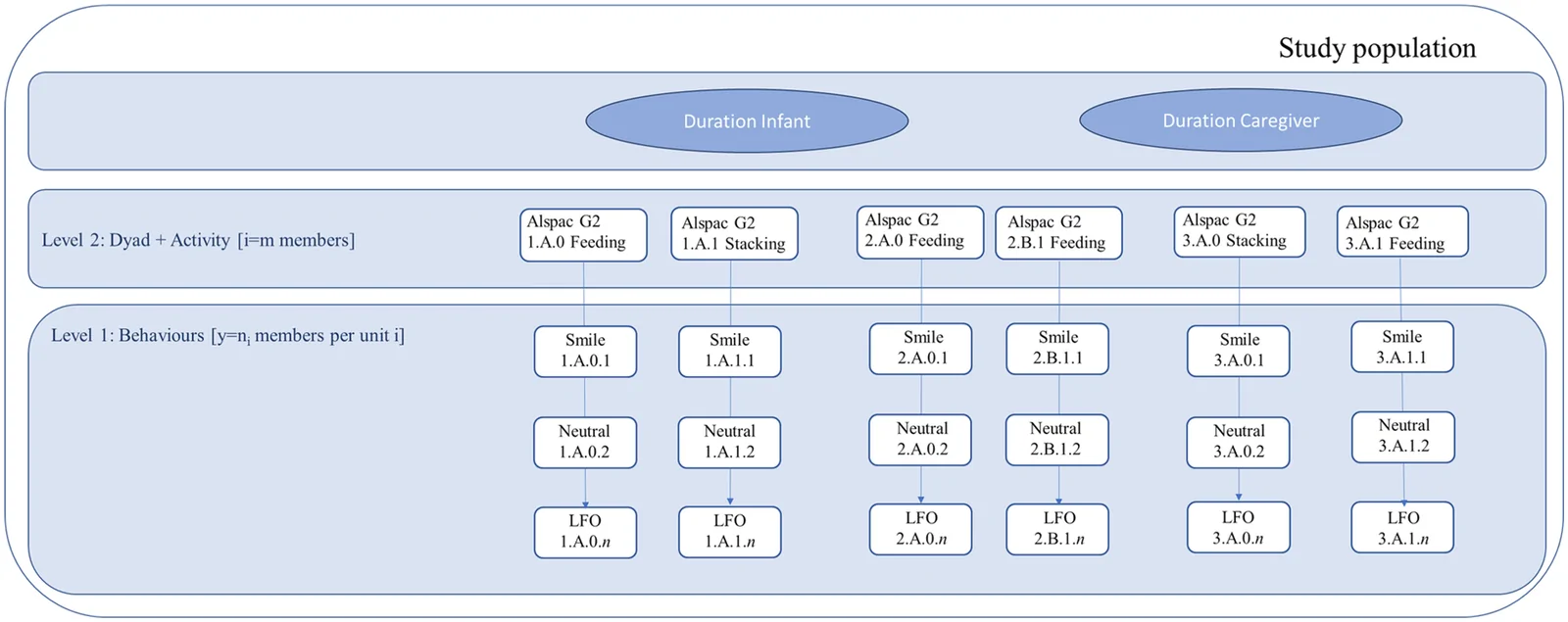
Hierarchical structure of the data used in the analyses. Note. This figure depicts the hierarchical data structure underlying the bivariate two-level models used to examine patterns of facial expressions and visual attention in infants and caregivers. Individual coded behavioural events (Level 1)—such as Smile, Neutral facial expression, or LFO (Look at Focus Object)—were nested within dyad–activity units (Level 2), defined by each parent–infant pair and the corresponding activity (e.g., feeding, stacking). The hierarchical notation (e.g., 1.A.0.1) indicates the family or dyad (first digit), caregiver (A = mother, B = father), activity, and sequential observation number (final digit). Infant and caregiver behavioural durations were estimated jointly within the same model, represented by the two upper boxes (“Duration Infant” and “Duration Caregiver”), corresponding to the two dependent variables of the bivariate model. The dependent variables were normalised proportions of total duration (n_pduration0 and n_pduration1), modelling the proportion of time spent in each behaviour relative to the total observation. Each behaviour was entered as a binary indicator, with the most frequent category serving as the reference group (“Face not visible” for facial expressions and “Look at Focus Object” for visual attention). The model thus accounted for the non-independence of behavioural observations within dyad–activity units and estimated the covariance between infant and caregiver behavioural durations. All coding followed the MHINT manual, which provides standardised operational definitions for facial expressions and visual attention. When multiple coders rated the same observation, data from the coder with the highest number of total coded observations were retained to maximise reliability.

Statistical analyses were conducted using Stata 17 (49), with multi-level models fitted in MLwiN (50). Figure 3 illustrates the hierarchical nature of the data. All analyses were pre-specified and exploratory (hypothesis-generating); no additional or *post-hoc* analyses were introduced based on observed results.

### Objective 1: patterns of visual attention and facial expressions in caregivers and infants

To characterise the total duration of parent and infant behaviours, we employed bivariate two-level models. Level 1 corresponded to individual behaviour observations nested within Level 2 units defined by each dyad–activity combination. Five nested models (Models 0–4) were modelled per behavioural group and mental health exposure, using restricted iterative generalised least squares (RIGLS), which performs well in small sample contexts. This resulted in 20 model sets in total: facial expression and visual attention behaviours were each modelled in relation to personality difficulties (SAPAS) and depressive symptoms (EPDS) (4 combinations × 5 models). These estimates were used as starting values for Markov Chain Monte Carlo (MCMC) estimation using Gibbs sampling, with default priors, a 1,000 iteration burn-in, and 10,000 iterations retained (thinning = 1).

We jointly modelled the proportion of time spent in each behaviour for infants and caregivers. The dependent variables were normalised proportion of total duration scores (i.e., n_pduration0 and n_pduration1) for infant and parent behaviours, respectively. These were computed by standardising the raw proportion of duration across observations to improve comparability and model convergence. To model behavioural durations, we used the most frequent category in each domain as the reference group: “Face not visible” for facial expressions and “Look at focus object” for visual attention. Each of the remaining behaviours was entered as a separate binary indicator, and model coefficients were estimated as the difference in mean duration of that behaviour compared with the reference category. Using the most common category as the reference facilitated comparability across models and ensured more stable estimates given the limited sample size.

### Objective 2: association between parental mental health and personality difficulties and the total duration of visual attention and facial expression

To explore associations between parental depressive symptoms and personality difficulties (measured by EPDS and SAPAS, respectively) and behavioural durations, we extended the bivariate two-level models used for descriptive characterisation by adding fixed-effect terms for standardised EPDS or SAPAS scores and their interactions with each non-reference behavioural indicator. Infant behaviour interactions were entered in Equation 1; parent behaviour interactions in Equation 2. EPDS and SAPAS scores were standardised (z-scores, M = 0, SD = 1), with higher scores reflecting worse mental health symptoms. Behaviours were modelled relative to the most frequent category within each domain, with “face not visible” serving as the reference for facial expressions and “look at focus object” for visual attention. Models were adjusted for the same set of covariates and retained the same nesting of observations within dyad–activity combinations. As in Objective 1, estimation used RIGLS for starting values followed by Markov Chain Monte Carlo with Gibbs sampling (burn-in = 1,000, 10,000 retained).

Model coefficients represent the proportional increase or decrease in behavioural duration per standard deviation increase in depressive symptoms (EPDS) or personality difficulties (SAPAS) score, relative to the respective reference category. Model fit statistics and diagnostics are reported in Supplementary Appendix, p.7 (48, 51).

Across both objectives, analyses were conducted within a Bayesian framework, and we therefore report posterior means together with 95% credible intervals (CrIs). Associations were considered to represent clear evidence when the 95% CrI excluded zero. Where CrIs included zero but the point estimates were consistent in direction across behaviours, we refer to these as suggestive findings. To aid interpretation of the standardised outcomes, we conducted linear regressions of the unstandardised proportion duration on the normalised proportion of duration scores. These analyses indicated that a one-unit increase in the normalised proportion of duration corresponded to approximately a 40-percentage point increase in raw behavioural duration. Given that most observations lasted five minutes (300 s), this equates to a difference of approximately 120 s. Accordingly, a coefficient of 0.25 would imply a 10-percentage point (or 30-second) difference in the duration of a given behaviour.

#### Sensitivity analyses

To assess potential bias in the complete case analyses, we compared summary statistics (frequencies, means, and standard deviations) of parent and infant behavioural durations between participants who completed the EPDS and/or SAPAS and those who did not. Demographic characteristics were also compared across these groups (see Table 1).

**Table 1:** Descriptives of demographic variables in participants who have complete the EPDS and/or the SAPAS and in those who have not.

Continuous variables	EPDS was not missing			EPDS was missing		
	*N*	Mean	SD	*N*	Mean	SD
Maternal age	73	26.16	2.08	24	26.79	2.17
Paternal age	55	28.64	4.82	18	28.70	2.62
Age child	73	6.88	0.93	24	7.79	2.64
Categorical variables	*N*	Mean		*N*	Mean	
Child sex						
Female	38	52.05		9	37.50	
Male	35	47.95		15	69.50	
Birth order						
First born	50	68.49		18	75.00	
Second born	20	27.40		5	20.83	
Third born	<5	4.11		<5	4.17	
Headcam						
Old	53	72.60		13	54.17	
New	20	27.40		11	45.83	
Main caregiver of the interaction						
Mother	61	83.56		20	83.33	
Father	12	16.44		<5	16.67	
Continuous variables	SAPAS was not missing			SAPAS was missing		
	*N*	Mean	SD	*N*	Mean	SD
Maternal age	83	26.43	2.04	14	25.64	2.44
Paternal age	63	28.89	4.54	9	27	2.73
Child age	83	7.05	1.17	14	7.42	3.08
Categorical variables	*N*	Mean		*N*	Mean	
Child sex						
Female	36	43.37		11	78.57	
Male	47	56.63		<5	21.43	
Birth order						
First born	58	69.88		10	71.43	
Second born	21	25.30		<5	28.57	
Third born	<5	4.82		–		
Headcam						
Old	55	66.27		11	78.57	
New	28	33.73		<5	21.43	
Main caregiver of the interaction						
Mother	70	84.34		11	78.57	
Father	13	15.66		<5	21.43	

EPDS, Edinburgh postnatal depression scale; SAPAS, standardised assessment of personality—abbreviated scale; *N*, number of participants; SD, standard deviation. “<5” indicates that fewer than five observations were present in a given category to preserve participant anonymity.

We also considered the possibility of non-random measurement error in the coding of visual attention due to headcam visibility constraints. Visual attention was coded using footage from both parent and infant headcams, where available. When only one camera provided usable footage, gaze direction could not be reliably cross-verified. This was captured using the modifier “Gaze direction—Not possible to code gaze direction”. To evaluate the impact of this limitation, we compared the variability in visual attention durations depending on whether gaze direction was observable or not (see Supplementary Appendix, p. 10 Supplementary STable 10).

## Results

### Demographic characteristics of the sample

The final analytic sample comprised 142 observations from 97 unique ALSPAC-G2 families, corresponding to 102 distinct parent–infant dyads. Five families contributed observations from more than one child. Among the 142 coded interactions, the primary caregiver was the biological mother in 105 observations (74%), and a biological father in 37 observations (26%). Although both mothers and fathers were included, the sample is predominantly maternal. 79 (81.4%) were ALSPAC-G1 participants, whilst the remaining participants were partners of participants previously enrolled in ALSPAC-G1. 10 (10%) families had both parents enrolled in ALSPAC-G1.

Of the 102 infants, 67 (66%) were first-born, and 72 (71%) were female. The median [IQR] age of the infants was 7 months [6–8 months]. The median [IQR] age of mothers was 26 years [25–28], and 28 years [26–30] for fathers. Parental depressive symptoms (EPDS) had a mean (SD) of 6.15 (4.46), and personality difficulties (SAPAS) had a mean (SD) of 2.17 (1.56). Each video lasted approximately five minutes (median duration = 5.00 min [IQR: 4.98–5.00).

Caregivers were asked to engage in typical at-home activities with their infants. The most frequently recorded activity was feeding (60.6%), followed by a structured stacking task (28.2%), free play (4.2%), and other routines such as bedtime or reading (7.0%). Examples of each activity type are described in the MHINT coding manual (52). Interactions took place in family homes, where siblings, pets, or other adults were occasionally present.

Sample characteristics for included vs. excluded families (e.g., due to missing data or attrition) are reported in Table 1.

### Objective 1: patterns of visual attention and facial expressions in caregivers and infants

Across participants with complete data on either the EPDS or the SAPAS, the most frequent facial expression code was “Face not visible,” with neutral, positive, and negative expressions observed less often. Negative facial expressions such as disgust and sadness were rare in both infants and caregivers. For visual attention, parents and infants directed most of their attention to the shared object of interaction (typically food or a toy), and less time attending to each other, distractions, or secondary caregivers.

In the complete case dataset of participants completing the EPDS, the most frequent code in the facial expression behaviours was “Face not visible” (*n* = 213, 24.80%), which was used as reference behaviour in our modelling strategy. Overall, the infants spent 14% less time [95% Credible Intervals (CrIs): 0%–44%] showing a disgusted face compared to the reference behaviour. Parents also showed shorter durations of disgust, negative facial expressions (e.g., sadness, discomfort, anger, fear), and woe face (empathic concern), relative to the reference behaviour (Supplementary STable 11). Infants also spent less time looking at the primary caregiver, at an object outside of the view of the camera, and at a distraction compared to the object focus of the interaction (i.e., reference group). The primary caregiver spent less time looking at the secondary caregiver or showing no visual attention compared to the reference.

In the complete case dataset of participants completing the SAPAS, the most frequent code in the facial expression behaviours was “Face not visible” (*n* = 234, 24.12%), followed by “Neutral/Alert” (*n* = 206, 21.24%), “Positive” (*n* = 162, 16.70%), and “Smile” (*n* = 143, 14.74%). Infants spent 14% (95%CrIs: −0.02–0.29) less time smiling compared to the reference behaviour (“face not visible”). Infants also spent 30% (95%CrIs: 10%–50%) less time looking at the caregiver compared to the time spent looking at the object focus of the interaction (e.g., food during feeding).

In the complete case samples (EPDS and SAPAS), the most frequent facial expression code was “*Face not visible”*, which was used as the reference category in all models. Among infants, facial expressions such as *disgust* and *negative affect* were observed less frequently than the reference. Parents also showed shorter durations of *disgust*, *negative expression*, and *woe face*.

For visual attention, infants spent less time looking at caregivers or distractions compared to the focus object of the interaction (e.g., food or toy). Caregivers, similarly, spent less time looking at the secondary caregiver or displaying no visual attention relative to the focus object.

### Objective 2: association between parental mental health and personality difficulties and the total duration of visual attention and facial expression

There was limited evidence for consistent associations between parental mental health and the total duration of facial expressions and visual attention behaviours. However, several exploratory associations were observed and are presented in full in Tables 1, 3–5 and Supplementary Appendix pp. 8–10.

**Table 2:** Unadjusted and adjusted associations between EPDS scores and facial expression durations in 117 dyads.

Interaction between EPDS score and each of the behaviours	Unadjusted models		Fully adjusted	
Infant facial expressions	Beta^a^	95% CrIs	Beta^b^	95% CrIs
Disgust	−0.12	−0.35, 0.11	−0.12	−0.34, 0.11
Negative	−0.10	−0.27, 0.06	−0.10	−0.27, 0.07
Neutral	−0.02	−0.13, 0.10	−0.02	−0.13, 0.09
Infant positive	−0.06	−0.18, 0.06	−0.06	−0.18, 0.07
Infant smile	0.15	0.00, 0.30	0.15	0.01, 0.30
None of the above	−0.02	−0.29, 0.26	−0.01	−0.29, 0.27
Caregiver facial expressions	Beta^a^	95% CrIs	Beta^b^	95% CrIs
Disgust	0.35	−0.12, 0.82	0.34	−0.12, 0.79
Mock surprise	−0.14	−0.28, 0.00	−0.14	−0.28, 0.00
Negative	0.24	−0.02, 0.50	0.24	−0.03, 0.51
Neutral	0.00	−0.12, 0.12	0.00	−0.12, 0.12
Positive	−0.01	−0.14, 0.11	−0.01	−0.14, 0.11
Smile	−0.01	−0.14, 0.12	−0.02	−0.14, 0.11
Surprise	−0.12	−2.84, 2.67	−0.17	−2.91, 2.54
“Woe” face	−0.49	−0.85, −0.12	−0.49	−0.85, −0.13
None of the above	−0.01	−0.17, 0.15	−0.01	−0.16, 0.16

Estimates are posterior means with 95% Credible Intervals (CrIs). Coefficients reflect the proportional difference in normalised total duration of each behaviour per 1-SD increase in EPDS, relative to the reference behaviour (“Look at focus object” for visual attention; “Face not visible” for facial expressions).

a Unadjusted model (exposure and outcome only).

b Fully adjusted model [child age, child sex, parental age, caregiver role, birth order, headcam type (pre- vs post–COVID-19), and activity]. CrIs that exclude 0 indicate clear evidence of association; CrIs including 0 are considered suggestive only when estimates are directionally consistent across related behaviours.

**Table 3:** Unadjusted and adjusted associations between SAPAS scores and facial expression durations in 119 dyads.

Interaction between SAPAS score and each of the behaviours	Unadjusted models		Fully adjusted	
Infant facial expressions	Beta^a^	95% CrIs	Beta^b^	95% CrIs
Disgust	−0.25	−0.46, −0.04	−0.25	−0.47, −0.03
Negative	−0.13	−0.29, 0.03	−0.14	−0.30, 0.03
Neutral	−0.02	−0.14, 0.10	−0.02	−0.13, 0.10
Infant positive	0.01	−0.13, 0.14	0.01	−0.14, 0.15
Infant smile	0.05	−0.12, 0.22	0.04	−0.13, 0.20
None of the above	−0.04	−0.28, 0.20	−0.05	−0.29, 0.20
Caregiver facial expressions	Beta^a^	95% CrIs	Beta^b^	95% CrIs
Disgust	−0.48	−0.95, −0.02	−0.50	−0.96, −0.02
Mock surprise	−0.14	−0.29, 0.01	−0.14	−0.28, 0.00
Negative	−0.41	−0.64, −0.16	−0.41	−0.64, −0.18
Neutral	−0.01	−0.13, 0.11	0.00	−0.12, 0.12
Positive	−0.02	−0.14, 0.11	−0.01	−0.14, 0.11
Smile	−0.03	−0.15, 0.10	−0.03	−0.15, 0.10
Surprise	−0.22	−0.62, 0.18	−0.23	−0.62, 0.17
“Woe” face	−0.60	−0.95, −0.24	−0.59	−0.95, −0.24
None of the above	−0.19	−0.37, 0.00	−0.19	−0.37, −0.01

Estimates are posterior means with 95% Credible Intervals (CrIs). Coefficients reflect the proportional difference in normalised total duration of each behaviour per 1-SD increase in SAPAS, relative to the reference behaviour (“Look at focus object” for visual attention; “Face not visible” for facial expressions).

a Unadjusted model (exposure and outcome only).

b Fully adjusted model [child age, child sex, parental age, caregiver role, birth order, headcam type (pre- vs post–COVID-19), and activity]. CrIs that exclude 0 indicate clear evidence of association; CrIs including 0 are considered suggestive only when estimates are directionally consistent across related behaviours.

**Table 4:** Unadjusted and adjusted associations between EPDS scores and visual attention behaviours in 112 dyads.

Interaction between EPDS score and each of the behaviours	Unadjusted models		Fully adjusted	
Infant visual attention	Beta^a^	95% CrIs	Beta^b^	95% CrIs
Look at caregiver 1	0.06	−0.01, 0.12	0.05	−0.01, 0.12
Look at caregiver 2	0.02	−0.10, 0.14	0.02	−0.11, 0.14
Look at distraction	0.08	−0.01, 0.18	0.08	−0.02, 0.17
Look at object outside of view	0.07	0.00, 0.14	0.07	0.00, 0.14
Look at other object	−0.02	−0.11, 0.06	−0.02	−0.11, 0.06
Look at other person	−0.13	−0.61, 0.38	−0.13	−0.62, 0.37
Look at other child (e.g., sibling)	0.29	0.16, 0.42	0.28	0.15, 0.41
No visual attention	0.00	−0.16, 0.15	0.00	−0.16, 0.15
Not possible to code visual attention	−0.01	−0.11, 0.09	−0.01	−0.12, 0.09
Caregiver visual attention	Beta^a^	95% CrIs	Beta^b^	95% CrIs
Look at caregiver 2	0.37	0.18, 0.56	0.37	0.18, 0.56
Look at distraction	0.06	−0.04, 0.16	0.06	−0.04, 0.16
Look at infant	0.03	−0.03, 0.09	0.03	−0.03, 0.09
Look at object outside of view	0.04	−0.02, 0.10	0.04	−0.03, 0.10
Look at other object	−0.04	−0.11, 0.03	−0.04	−0.11, 0.03
Look at other person	0.04	−0.41, 0.48	0.04	−0.41, 0.48
Look at other child (e.g., sibling)	−0.02	−0.18, 0.14	−0.02	−0.17, 0.14
No visual attention	0.18	−0.09, 0.46	0.18	−0.10, 0.46
Not possible to code visual attention	0.05	−0.05, 0.14	0.05	−0.05, 0.15

Estimates are posterior means with 95% Credible Intervals (CrIs). Coefficients reflect the proportional difference in normalised total duration of each behaviour per 1-SD increase in EPDS, relative to the reference behaviour (“Look at focus object” for visual attention; “Face not visible” for facial expressions).

a Unadjusted model (exposure and outcome only).

b Fully adjusted model [child age, child sex, parental age, caregiver role, birth order, headcam type (pre- vs post–COVID-19), and activity]. CrIs that exclude 0 indicate clear evidence of association; CrIs including 0 are considered suggestive only when estimates are directionally consistent across related behaviours.

**Table 5:** Unadjusted and adjusted associations between SAPAS scores and visual attention behaviours in 119 dyads.

Interaction between SAPAS score and each of the behaviours	Unadjusted models		Fully adjusted	
Infant visual attention	Beta^a^	95% CrIs	Beta^b^	95% CrIs
Look at caregiver 1	0.03	−0.04, 0.09	0.02	−0.04, 0.09
Look at caregiver 2	−0.02	−0.12, 0.09	−0.02	−0.12, 0.08
Look at distraction	0.06	−0.05, 0.17	0.06	−0.05, 0.17
Look at object outside of view	0.01	−0.06, 0.08	0.01	−0.06, 0.07
Look at other object	0.07	0.00, 0.15	0.07	−0.01, 0.15
Look at other child (e.g., Sibling)	0.12	0.02, 0.21	0.11	0.02, 0.21
No visual attention	0.04	−0.12, 0.19	0.04	−0.12, 0.19
Not possible to code visual attention	0.02	−0.08, 0.13	0.02	−0.09, 0.12
Caregiver visual attention	Beta^a^	95% CrIs	Beta^b^	95% CrIs
Look at caregiver 2	−0.04	−0.18, 0.09	−0.04	−0.17, 0.09
Look at distraction	−0.06	−0.17, 0.05	−0.06	−0.17, 0.05
Look at infant	−0.03	−0.10, 0.05	−0.03	−0.10, 0.05
Look at object outside of view	−0.06	−0.14, 0.02	−0.06	−0.14, 0.02
Look at other object	−0.05	−0.13, 0.03	−0.05	−0.13, 0.03
Look at other person	0.04	−0.45, 0.50	0.03	−0.46, 0.51
Look at other child (e.g., sibling)	−0.06	−0.16, 0.05	−0.06	−0.17, 0.05
No visual attention	−0.01	−0.25, 0.24	−0.01	−0.25, 0.24
Not possible to code visual attention	−0.05	−0.16, 0.06	−0.05	−0.16, 0.06

Estimates are posterior means with 95% Credible Intervals (CrIs). Coefficients reflect the proportional difference in normalised total duration of each behaviour per 1-SD increase in SAPAS, relative to the reference behaviour (“Look at focus object” for visual attention; “Face not visible” for facial expressions).

a Unadjusted model (exposure and outcome only).

b Fully adjusted model [child age, child sex, parental age, caregiver role, birth order, headcam type (pre- vs post–COVID-19), and activity]. CrIs that exclude 0 indicate clear evidence of association; CrIs including 0 are considered suggestive only when estimates are directionally consistent across related behaviours.

Among participants with EPDS data, higher depressive symptoms were associated with shorter durations of mock surprise [–0.14, 95% Credible Intervals (CrI): −0.28–0.00] and woe face (–0.49, 95% CrI: −0.85 to −0.13) in caregivers. Weak evidence also suggested longer durations of negative facial expressions (0.24, 95% CrI: −0.03–0.51), although the CrI crossed zero. Infants of caregivers reporting more depressive symptoms spent approximately 15% more time smiling (95% CrI: 0.01–0.30) (Table 2).

Among participants with SAPAS data, higher personality difficulty scores were associated with shorter durations of mock surprise (−0.14, 95% CrI: −0.28–0.00) and woe face (−0.59, 95% CrI: −0.95 to −0.24). However, in contrast to the EPDS results, caregivers with higher SAPAS scores, indicating more personality difficulties, also showed shorter durations of negative affect (–41%, 95% CrI: −64 to −18) and disgust (–50%, 95% CrI: −96 to −2) (Table 3).

For visual attention behaviours, caregivers with higher depressive symptoms spent more time looking at the secondary caregiver (37% more, 95% CrI: 18–56). Infants of these caregivers spent more time looking at objects outside the camera's view (7%, 95% CrI: 0–14) and at other people present (28%, 95% CrI: 15–41), such as siblings or other relatives (Table 4). In the SAPAS models, few associations emerged, although infants of parents with higher personality difficulties spent more time looking at another child (11%, 95% CrI: 2–21) (Table 5).

Further details on the random part of the model and model diagnostics and fit are provided in the Supplementary Appendix, pp. 8–10.

#### Sensitivity analyses

To assess the impact of missing mental health data, we compared behaviour durations among those who completed the EPDS or SAPAS to those who did not (Supplementary Appendix, 10). Some differences were observed, indicating that bias from complete case analysis cannot be ruled out. Demographic comparisons showed no systematic differences between participants with and without mental health data.

We also examined the potential influence of measurement error due to headcam limitations. Specifically, we tested whether behaviour durations differed depending on whether gaze direction could be coded using one or both cameras. No systematic differences in means or variability were found, suggesting no strong pattern of bias based on headcam visibility.

## Discussion

This study provides novel evidence that parent–infant interactions can be captured in ecologically valid home settings within a longitudinal cohort study using wearable, first-person cameras. Using bivariate multilevel models, we explored whether parental depressive symptoms and personality difficulties were associated with the total duration of facial expressions and visual attention in parents and infants.

While mental health symptoms in this community-based sample were generally mild, we observed some tentative associations between parental mental health in pregnancy (or preconception) and different durations of facial expression and visual attention behaviours. Higher depressive symptoms and personality difficulties were linked to shorter durations of *woe face* expressions—an affective marker of empathic mirroring. Indeed, showing a “woe” face has been considered an empathic response to distress and negative facial expressions (8, 12). Parents with higher depressive symptoms also showed reduced *mock surprise* and marginally increased negative facial expressions. Since we did not assess the temporal sequences of these behaviours in this study, we cannot establish whether the negative facial expression is an attempt at mirroring expressions in the infant and/or an expression of frustration and/or a response to a stressful situation. However, we could speculate that the ability to imitate or mirror and, in general, connect with the infant's negative emotions may be reduced in participants with mental health problems who may have more difficulties in managing their own emotional distress and, thus, find it harder to contain others’ negative emotions (53). This would align with findings that individuals with depression withdraw their attention faster from stimuli of distress (27) and neuroimaging findings that report that depressed mothers show dampened neural activation of mirror neurons when imitating or empathising with their child's facial expressions (63) or their infant's cry (54).

The timing of mental health assessment is an important consideration. In this study, depressive symptoms and personality difficulties were measured during the prenatal or preconception period, several months before the observed parent–infant interactions. Measuring symptoms before the interaction reduces the risk of reverse causality (e.g., difficult interactions exacerbating parental symptoms), but it also means that symptoms may have changed during this interval and may not fully capture parents’ mental health at the time of observation. Nonetheless, associations between earlier symptoms and later interaction quality suggest that difficulties can exert longer-term effects on relational dynamics. This interpretation is consistent with meta-analytic evidence indicating that while current depression has the strongest impact on parenting—particularly negative behaviours—residual effects of prior depression also persist across domains of sensitivity and engagement (21). These findings underscore the importance of supporting parents both during and after depressive episodes, as difficulties may continue to influence parent–infant interaction and infant socioemotional development.

Mirroring negative emotions and attuning to child distress may be more arousing for participants with mental health difficulties. When the parent was more depressed, we found some evidence that they spent more time looking at another caregiver along with some evidence that their infant looked at the primary caregiver, at objects outside of the footage focus, and at another child (most likely their sibling) for longer. Both the child and parent's patterns of attention toward other people in the room may indicate that when parents experience more symptoms of mental health difficulties they refer to the other caregiver more (e.g., seeking support or disengaging from the child) and that the child similarly may search for people other than the caregiver for potential social referencing (55).

Interestingly, infants of more depressed caregivers smiled more, consistent with prior research suggesting infants of more depressed parents spent more time smiling (56). While effect sizes were small and uncertainty was high, this may reflect adaptive infant strategies or altered affective signalling.

### Strengths

Although exploratory, this study has several strengths. First, it included both mothers and fathers from a prospective longitudinal cohort. Much of the existing literature focuses exclusively on maternal mental health and mother–infant interactions; inclusion of fathers, albeit in smaller numbers, broadens the scope of inquiry. Second, behavioural coding was performed by trained raters who were blinded to parental mental health status, reducing the risk of bias in outcome measurement. Third, child and caregiver behaviours were modelled simultaneously using bivariate multilevel models, allowing more efficient use of the data and enabling direct comparisons across dyad members (57).

Fourth, interactions were recorded using wearable, first-person cameras in the home environment without the presence of a researcher. This approach enhances ecological validity by capturing a broader range of naturalistic behaviours—including those less likely to occur under observation, such as caregiver expressions of frustration or self-conscious behaviour (34). The use of a first-person perspective offers additional methodological advantages. Compared to traditional third-person recording, wearable cameras provided closer and clearer views of participants’ faces, improving the accuracy of coding facial expressions and potentially supporting the future use of automated facial recognition tools (e.g., Noldus FaceReader®) (58).

From a clinical perspective, this method may have valuable applications in parenting interventions. Video-feedback approaches, which have been shown to improve parental sensitivity (59), often rely on third-person recordings taken by practitioners. First-person footage could enhance these interventions by presenting parents with a more intimate and representative view of their interactions, especially in families where traditional filming might inhibit natural behaviour. Prior research has found that when recorded without an observer, parents—particularly those with mental health difficulties—are more likely to display both socially desirable and undesirable behaviours (e.g., singing, criticism). These authentic moments may offer richer material for strength-based feedback (36). Given evidence that parents with mental health difficulties may be slower at learning from infant social feedback (60), first-person footage may foster perspective-taking and improve empathetic and reflective skills.

### Limitations

Several potential limitations should be noted. First, findings should be interpreted with caution as we lacked statistical power due to small sample and moderate levels of missingness in our exposures. Although comparisons between participants with and without complete mental health data suggested only minor differences in behaviour duration, we did not apply multiple imputation due to the complexity of the multilevel structure.

Second, limited availability of covariates prevented full adjustment for confounding, which may bias estimates. Third, selection bias is a potentially severe limitation of this study. Prior work has shown that ALSPAC-G1 participants who enrolled their children in G2 differ systematically from those who did not—particularly in education and study engagement (40). As fewer than 35% of those invited participated and only 63% of these were included in analyses, generalisability may be limited. Without access to mental health data on non-participants, we were unable to apply inverse probability weighting to address this bias.

Fourth, misclassification of behaviours is possible. Although coders were blinded and used event-based schemes with objective criteria, some behaviours may have been affected by camera angle or occlusion. We examined whether the codability of gaze (i.e., whether gaze direction could be determined) affected behavioural coding and found no consistent pattern of error across behaviours.

Fifth, behavioural data were skewed, with many zero values for less frequent behaviours. This may have led to biased estimates in standard linear models. Although not explored here, future work should consider two-part or hurdle models to better accommodate zero-inflated data structures (61).

Finally, our analyses focused solely on total duration of behaviours. Temporal dynamics—including synchrony, sequencing, and responsiveness—may offer additional insights into parent–infant interaction patterns and should be the focus of future analyses.

### Future directions and conclusions

Analysing the temporal sequencing of parent–infant interactions may provide further insight into aspects of relational functioning that are disrupted by parental mental health difficulties. A key aim for future work is to build on these findings to strengthen available support for parents and families, with the goal of preventing or reducing internalising difficulties in children. Integrating synchronised dyadic first-person footage—capturing simultaneous close-up views of both infant and parent facial expressions—into parenting programmes, such as video-feedback interventions, may enhance their effectiveness by helping parents to view the interaction from their infant's perspective.

## Data Availability

The data analyzed in this study is subject to the following licenses/restrictions: The steps below highlight how to apply for access to the data included in the data note and all other ALSPAC data: (1) Please read the ALSPAC access policy (http://www.bristol.ac.uk/media-library/sites/alspac/documents/researchers/data-access/ALSPAC_Access_Policy.pdf) which describes the process of accessing the data and samples in detail, and outlines the costs associated with doing so. (2) You may also find it useful to browse our fully searchable research proposals database (https://proposals.epi.bristol.ac.uk/?q=proposalSummaries), which lists all research projects that have been approved since April 2011. (3) Please submit your research proposal (https://proposals.epi.bristol.ac.uk/) for consideration by the ALSPAC Executive Committee. You will receive a response within 10 working days to advise you whether your proposal has been approved. Requests to access these datasets should be directed to https://proposals.epi.bristol.ac.uk/.
