# Correction: Longitudinal associations between parental mental health and the duration of visual attention and facial expressions during at-home parent–infant interactions: a UK birth cohort study

**DOI:** 10.3389/frcha.2026.2016274

**Published:** 2026-10-26

**Authors:** Ilaria Costantini, Daphne Kounali, Iryna Culpin, Marc H. Bornstein, Rebecca M. Pearson

**Affiliations:** 1 Division of Psychiatry, University College London, London, United Kingdom; 2 Centre for Academic Mental Health, Population Health Sciences, University of Bristol, Bristol, United Kingdom; 3 Oxford Clinical Trials Research Unit, Centre for Statistics in Medicine, Nuffield Department of Orthopaedics, Rheumatology and Musculoskeletal Sciences, University of Oxford, Oxford, United Kingdom; 4 Florence Nightingale Faculty of Nursing, Midwifery and Palliative Care, Department of Child and Family Health, King’s College London, London, United Kingdom; 5 Eunice Kennedy Shriver National Institute of Child Health and Human Development, Bethesda, MD, United States; 6 Institute for Fiscal Studies, London, United Kingdom; 7 Department of Population Health Sciences, Bristol Medical School, University of Bristol, Bristol, United Kingdom; 8 Faculty of Health and Education, School of Psychology, Manchester Metropolitan University, Manchester, United Kingdom

**Keywords:** ALSPAC, parent-infant interactions, depression, personality difficulties, headcams

Affiliation ¨ 4. United Nations Children's Fund (UNICEF), New York, NY, United States¨ was erroneously included in the paper. This affiliation has now been removed for authors Iryna Culpin and Marc H. Bornstein.

Affiliation ¨4. Florence Nightingale Faculty of Nursing, Midwifery and Palliative Care, Department of Child and Family Health, King's College London, London, United Kingdom¨ was omitted from the paper. This affiliation has now been added for author Iryna Culpin.

The original version of this article has been updated.

