# Supplementary material for: Longitudinal associations between parental mental health and the duration of visual attention and facial expressions during at-home parent–infant interactions: a UK birth cohort study
Source: Front Child Adolesc Psychiatry. 2025 Oct 27;4:1638234. doi: 10.3389/frcha.2025.1638234 (PMC12597733; doi:10.3389/frcha.2025.1638234)
Supplement: Supplementary file 1 [file Datasheet1.docx]

# **Appendix**

# **Longitudinal associations between parental mental health and duration of visual attention and facial expression during at-home parent-infant interactions: a UK birth cohort study**

Ilaria Costantini^1,2^, PhD; Daphne Kounali^2,3^, PhD; Iryna Culpin^2,6^, PhD; Marc H. Bornstein^4,5,6^, PhD, Rebecca M. Pearson^2,7,8^, PhD.

1 Division of Psychiatry, University College London, London, United Kingdom.

2 Centre for Academic Mental Health, University of Bristol, Oakfield House, Bristol, United Kingdom.

3 Oxford Clinical Trials Research Unit, Centre for Statistics in Medicine, Nuffield Department of Orthopaedics, Rheumatology and Musculoskeletal Sciences, University of Oxford, Oxford, UK

4 *Eunice Kennedy Shriver* National Institute of Child Health and Human Development, Bethesda, MD USA

5 Institute for Fiscal Studies, London, UK

6 UNICEF, New York, NY, USA

7 Department of Population Health Sciences, Bristol Medical School, University of Bristol, Bristol, UK

8 School of Psychology, Faculty of Health and Education, Manchester Metropolitan University, Manchester, UK

Contents

[**Appendix** 1](#_Toc209697796)

[**Longitudinal associations between parental mental health and duration of visual attention and facial expression during at-home parent-infant interactions: a UK birth cohort study** 1](#_Toc209697797)

[**Supplement Methods** 4](#_Toc209697798)

[Further details on recruitment into the study 4](#_Toc209697799)

[Video recording procedures using the headcams 4](#_Toc209697800)

[Old and new headcams 4](#_Toc209697801)

[Pre-Covid procedure 4](#_Toc209697802)

[Covid and Post-Covid procedures: Data collection 5](#_Toc209697803)

[Development of MHINT Manual 7](#_Toc209697804)

[Further details on outcome 7](#_Toc209697805)

[Further details on model fit and selection 7](#_Toc209697806)

[Sample characteristics 8](#_Toc209697807)

[Results on model fit and model diagnostics 8](#_Toc209697808)

[Further details on final selected model 8](#_Toc209697809)

[Associations Between Mental Health and Behavioural Outcomes 9](#_Toc209697810)

[Sensitivity analyses 10](#_Toc209697811)

[**Supplementary Tables** 19](#_Toc209697812)

[Descriptive and random effects of the models 22](#_Toc209697813)

[**References** 35](#_Toc209697814)

[STable 1. Descriptive statistics for total duration of infant and parent facial expressions and visual attention behaviours, comparing participants with and without missing mental health measures (EPDS and SAPAS) in 97 families. 19](#_Toc211252298)

[STable 2. Adjusted model of the EPDS on visual attention behaviours in infants (first part of the equation) and parents (second part of the equation). 22](#_Toc211252299)

[STable 3. Adjusted model of the EPDS on total duration of facial expressions in infants (first part of the equation) and parents (second part of the equation). 23](#_Toc211252300)

[STable 4. Adjusted model of SAPAS on total duration of visual attention in infants (first part of the equation) and parents (second part of the equation). 24](#_Toc211252301)

[STable 5. Adjusted model of SAPAS on total duration of facial expression in infants (first part of the equation) and parents (second part of the equation). 25](#_Toc211252302)

[STable 6. Random effects component of all the models presented. 26](#_Toc211252303)

[STable 7. Model fit diagnostics for all included models. 28](#_Toc211252304)

[STable 8. Adjusted model 5 models of facial expressions with the EPDS and the SAPAS in complete case analyses (participants with both the EPDS and the SAPAS). 30](#_Toc211252305)

[STable 9. Adjusted model 5 models of facial expressions with the EPDS and the SAPAS in complete case analyses (participants with both the EPDS and the SAPAS). 31](#_Toc211252306)

[STable 10. Frequencies, means, and standard deviations of child and parent normalised score of the proportion of total duration of visual attention by its modifiers (i.e., gaze direction). 33](#_Toc211252307)

[SFigure 1. Headcam and photoframe instructions. 10](#_Toc209697825)

[SFigure 2. This figure illustrates the most relevant coding schemes and theoretical influences on the development of the MHINT manual. 11](#_Toc209697826)

[SFigure 3. Histograms of mental health measures used in this study. 12](#_Toc209697827)

[SFigure 4. Histograms of the normalised proportion of total duration score in the EPDS complete case dataset. 13](#_Toc209697828)

[SFigure 5. Histograms of the normalised proportion of total duration score in the SAPAS complete case dataset. 14](#_Toc209697829)

[SFigure 6. Residuals from Model 4: EPDS and facial expression. 15](#_Toc209697830)

[SFigure 7. Residual from Model 4: EPDS and visual attention. 16](#_Toc209697831)

[SFigure 8. Residuals from Model 4: SAPAS and facial expression. 17](#_Toc209697832)

[SFigure 9. Residuals from Model 4: SAPAS and visual attention 18](#_Toc209697833)

# **Supplement Methods**

## Further details on recruitment into the study

Initially, biological fathers and maternal partners were invited to participate in the headcams study indirectly, via an invitation sent to the mother when their child enrolled in ALSPAC-G2. On 22 July 2019, with additional funding from the Wellcome Trust, a dedicated research clinic—*Focus on Fathers*—was launched. This clinic enabled direct recruitment of fathers when their ALSPAC-G2 child reached 6 months of age, inviting them to take part in a range of assessments, including the headcams study.

### Video recording procedures using the headcams

To maximise ecological validity, parent–infant interactions were recorded in participants’ own homes, without the presence of a researcher. This approach reduces social desirability bias and supports the capture of more naturalistic behaviour (Lee *et al.*, 2017).

### Old and new headcams

Two generations of head-mounted cameras were used. The ‘old’ headcams were Bogdan Digital Spy Hidden Camera DVR video recorders, costing approximately £25 each, with an additional £5 for SD card storage. These cameras recorded in AVI format at 720×480 resolution, 30 frames per second, and offered a field of view of approximately 60 degrees. Cameras were mounted on soft headbands and worn by both the parent and the infant. Figure 1 presents example footage from a consenting family, illustrating the perspectives captured.

The ‘new’ headcams, introduced post-Covid, were WearCam devices manufactured by Ucam247. These cameras offered improved technical specifications, including 1280×720 resolution, a wider 85-degree field of view, and the same 30 frames per second rate. A formal comparison of the performance of these new devices has been reported elsewhere (Skinner *et al.*, 2022).

## Pre-Covid procedure

Video and audio recordings of mother–infant and father–infant interactions were captured using two wearable cameras (WCs) and an additional third-person camera concealed within a photo frame. The wearable cameras, mounted on headbands, were worn by both the parent and the infant, allowing for two simultaneous first-person perspectives during each recorded session.

Quality control and security checks were carried out by ALSPAC fieldworkers before making the data available. Separate headcam footage from both the parent and infant cameras was synchronised by their audio wavelength using Premiere Pro software (<https://www.adobe.com/uk/products/premiere.html>). Parents were provided with fully charged cameras and instructed to record naturalistic mealtime and play interactions at home. Detailed procedural guidance, including setup instructions for both headcams and the photo frame camera, is available in **SFigure 1**.

## Covid and Post-Covid procedures: Data collection

Prior to participation, informed consent was obtained by trained ALSPAC fieldworkers. Consent was recorded electronically via the REDCap system; where electronic completion was not feasible, paper forms were used as a backup. Fieldworkers were fully trained in all relevant procedures, including both virtual and in-clinic protocols, and were familiar with the technical requirements of the headcam equipment.

For each participating family, a participant pack was prepared and labelled with the participant ID. The pack included:

- Two or three (if partner also takes part) fully charged and successfully tested head cameras were provided together with consent forms (one for each participant) at clinic visit
- Instruction sheet
- Activity recording schedule sheet (labelled with participant ID)
- Consent 21 (labelled with participant ID)
- Stacking toy (Clinic)

Fieldworkers ensured that the child was at least 6 months of age on the day of participation, as younger infants may not have been developmentally suited to the tasks (e.g., sitting in a high chair for mealtimes).

#### During the visit (virtual and clinic)

The fieldworker made sure that both Study Participant (SP) and their partners, if they agreed to take part into the study, had read the information booklet, had no further questions, and had correctly completed the e-consent page for both parent and child wearing the head cams (if the participant had other children who may appear in the recordings, it was ensured that the number of children was indicated).

Then, the process of recording a session and the instruction document which included visual representation of this were shown to the SP. At the end of the session the SP was provided with the bag containing the required equipment or was informed that the equipment would be sent in the virtual clinic equipment box.

#### Demonstrating recording and ending a session (virtual and clinic)

For virtual clinics, at the measurement session, a fieldworker used a camera to show participants how to correctly use the cameras, including how to turn it on and off, how to record a session, how to position it on themselves and their infant so that it did not obstruct the view but was also comfortable to wear.

#### Introducing the activity schedule

The SP were asked to record three sessions for as long as they usually would at specified times with the following specified activities:

1. At breakfast
2. At lunch
3. Stacking task (Clinic) Childs own activity type toy (Virtual)

They were instructed to write down the following:

- Start and end time of the recording
- Describe the activities during the session, for example: child sat in high chair and mum gave food whilst sitting at the table or child sat on mat and played with puzzle games (this is due to the narrow field of view of the camera which can sometimes make it hard to identify the exact nature of the activity)
- To cross the void box if they did not want a recording viewed

The SP was asked to record for the entire length of the activity, as the approved study was interested in all behaviours and reactions, and to agree on a suitable collection date with the participant and if appropriate liaise with COCO90s casual collection drivers or field workers to ensure cameras were collected.

#### After the visit (Clinic and Virtual)

Participants received a reminder the day before the agreed collection date. Fieldworkers retrieved all equipment and any completed consent forms. Data collection progress was tracked in REDCap and Arcadia systems. If data were successfully returned, participants received a voucher via Arcadia.

#### Virtual Clinic Procedure Only

In virtual clinics, equipment was shipped directly to participants' homes. Consent was obtained and documented electronically via REDCap. A Covid-specific safety protocol was established to support safe delivery and usage of equipment, including detailed written instructions for headcam operation.

During this period, the research team also collaborated with Kinneir Dufort (KD), a Bristol-based design consultancy specialising in medical and digital technologies, to refine the headcam hardware for enhanced usability and data quality. As a result, all post-Covid data collection used the upgraded wearable devices, and the third-person ‘photo frame’ camera was discontinued. An overview of the stages involved in the improvement of the headcams has been published previously

## Development of MHINT Manual

The MHINT coding manual was developed through a combination of top-down and bottom-up approaches. The top-down component was informed by theoretical frameworks and established coding systems (see **SFigure 2**). Of special relevance to the development of the MHINT manual was the work of Marc Bornstein (Bornstein, no date) and Beatrice Beebe (Beebe *et al.*, 2010) pertaining to attention to the environment and structure of the coding scheme (such as the use of priority rules), and facial expressions and gaze direction behaviours, respectively. The bottom-up development involved piloting the coding scheme on a subset of ALSPAC recordings. During this process, additional behaviour codes were incorporated to reflect interactional nuances not captured in existing systems. For instance, new codes were added for infant visual attention directed toward siblings or pets, which emerged as relevant in the naturalistic home environment.

## Further details on outcome

Each behavioural category (e.g., facial expressions, visual attention) was coded as a mutually exclusive and exhaustive stream, with the addition of modifiers to capture more granular variation. For example, within the visual attention category, modifiers allowed coders to specify gaze direction and whether the infant and caregiver were engaged in joint or divergent focus.

Whenever a modifier was applicable, both the behavioural code and its corresponding modifier were recorded. For this study, we focused on facial expression and visual attention behaviours for both infants and caregivers. The complete MHINT coding manual, which includes definitions, examples, and downloadable templates for use in Noldus Observer XT® software, is available online (Costantini et al., n.d.-a).

## Further details on model fit and selection

Model selection was informed by a combination of theoretical rationale, model fit statistics, and comparisons of deviance information criteria (**STable 8**). To assess consistency across exposure measures, we also fitted models in the subsample of participants with complete data on both the EPDS and SAPAS (**STable 9** and **STable 10**). However, to retain statistical power and reduce bias due to missing data, the primary analyses focused on separate complete case samples for each mental health measure.

**Supplement Results**

## Sample characteristics

**STable 1** presents descriptive comparisons between participants who completed the EPDS and SAPAS measures and those who did not. Variables compared include maternal and paternal age, child age, child sex, birth order, and type of headcam used. Overall, demographic differences between groups were minor. However, some variation was observed in child sex and headcam type. These differences suggest that missingness in mental health data is unlikely to have introduced substantial bias into the analyses.

## Results on model fit and model diagnostics

Model fit was assessed using the Deviance Information Criterion (DIC). For both EPDS and SAPAS models, DIC values were not substantially reduced when interaction terms between behaviours and mental health scores were included, as compared to simpler models (**STable 8**). Both the unadjusted (model 3) and the adjusted models (model 4) did not markedly improve compared to simpler models and, in some instances, they worsened. This may indicate that these measures are not explaining the data better than simply adding the mental health measures as covariates, as further illustrated by the little evidence of interaction between the mental health measures and the behaviours. However, the behaviour of the DIC could also relate to the lack of model calibration due to not fully accounting for the structural zeros, thereby we decided to present the findings from these models anyway, as they were developed based on a-priori hypotheses.

Distributions of residuals from the random effects of the first level (i.e., behavioural level) were skewed at the lower end of the distribution, this indicates that the effect estimates obtained from those behaviours that had shorter duration may be unreliable. In contrast, distributions of residuals from the random effects at the second level (i.e., at the dyad-activity level) were (approximately) normally distributed. This indicates that we should have been able to make accurate inferences about specific group effects. More work is needed to improve model calibration to account for structural zeros that are considered informative and should be explicitly modelled (such in two-part models)(Belotti *et al.*, 2015).

## Further details on final selected model

In order to appropriately compare the estimates between the EPDS and the SAPAS models, we ran the final selected model (i.e., interactions between mental health measures and behaviours and with covariates) in those participants who had completed both the EPDS and the SAPAS. However, we decided not to present these analyses only as secondary analyses as they reduce further statistical power by including only participants who completed both measures. The effect estimates, presented in **STable 9** and **STable 10**, are largely comparable to those found in the models presented in the main text, even though a slight increase in imprecision (i.e., wider credible intervals) is observable. These analyses were conducted as secondary analyses to ensure comparability but were not used as the primary analyses due to concerns regarding reduced statistical power.

## Associations Between Mental Health and Behavioural Outcomes

**STables 3** to **6** present results from adjusted models examining the association between mental health measures (EPDS and SAPAS) and infant and parent behaviours. **STable 3** reports findings from the model estimating associations between EPDS scores and visual attention behaviours. In this model, there was limited evidence of strong associations, although reduced caregiver looking at distraction and increased caregiver looking at the infant were among the more notable observations.

**STable 4** presents the results of the adjusted model investigating EPDS scores in relation to total duration of facial expressions. This analysis indicated that higher EPDS scores were associated with shorter durations of certain parental facial expressions, particularly disgust, mock surprise, negative affect, and ‘woe’ face, suggesting some evidence of altered affective expression associated with depressive symptoms.

**STable 5** summarises the adjusted model estimating associations between SAPAS scores and visual attention behaviours. Higher SAPAS scores were associated with reduced caregiver attention to distractions and increased attention towards siblings, although credible intervals indicated that the precision of these estimates was modest.

Similarly, **STable 6** reports findings from the adjusted model assessing associations between SAPAS scores and facial expressions. Higher SAPAS scores were associated with shorter durations of negative facial expressions and 'woe' face expressions, particularly in parents, with some evidence also observed in infant behaviours. Together, these results suggest that personality difficulties may be linked to alterations in patterns of affective display during parent–child interactions.

At the dyad and activity level, we found that the correlation of the parent and child total duration of behaviours is positive, which means that when a parent has longer duration of a particular behaviour, the child would also spend more time engaging in that behaviour on average (**STable 7**).

## Sensitivity analyses

To assess the potential for measurement error due to limited visibility of gaze, we examined whether variability in the duration of visual attention behaviours differed when gaze direction could be directly coded versus when it was inferred from a single headcam perspective. **STable 11** presents standard deviations for each condition. While some minor differences were observed, no systematic patterns emerged, suggesting that gaze visibility did not meaningfully bias behavioural duration estimates.

In **STable 2**, we compared the total duration of infant and caregiver facial expressions and visual attention behaviours across participants with and without EPDS or SAPAS data. These analyses indicated generally comparable behaviour patterns, again supporting the assumption that missing mental health data did not substantially affect behavioural distributions.

**Supplementary Figures**

SFigure 1. Headcam and photoframe instructions.


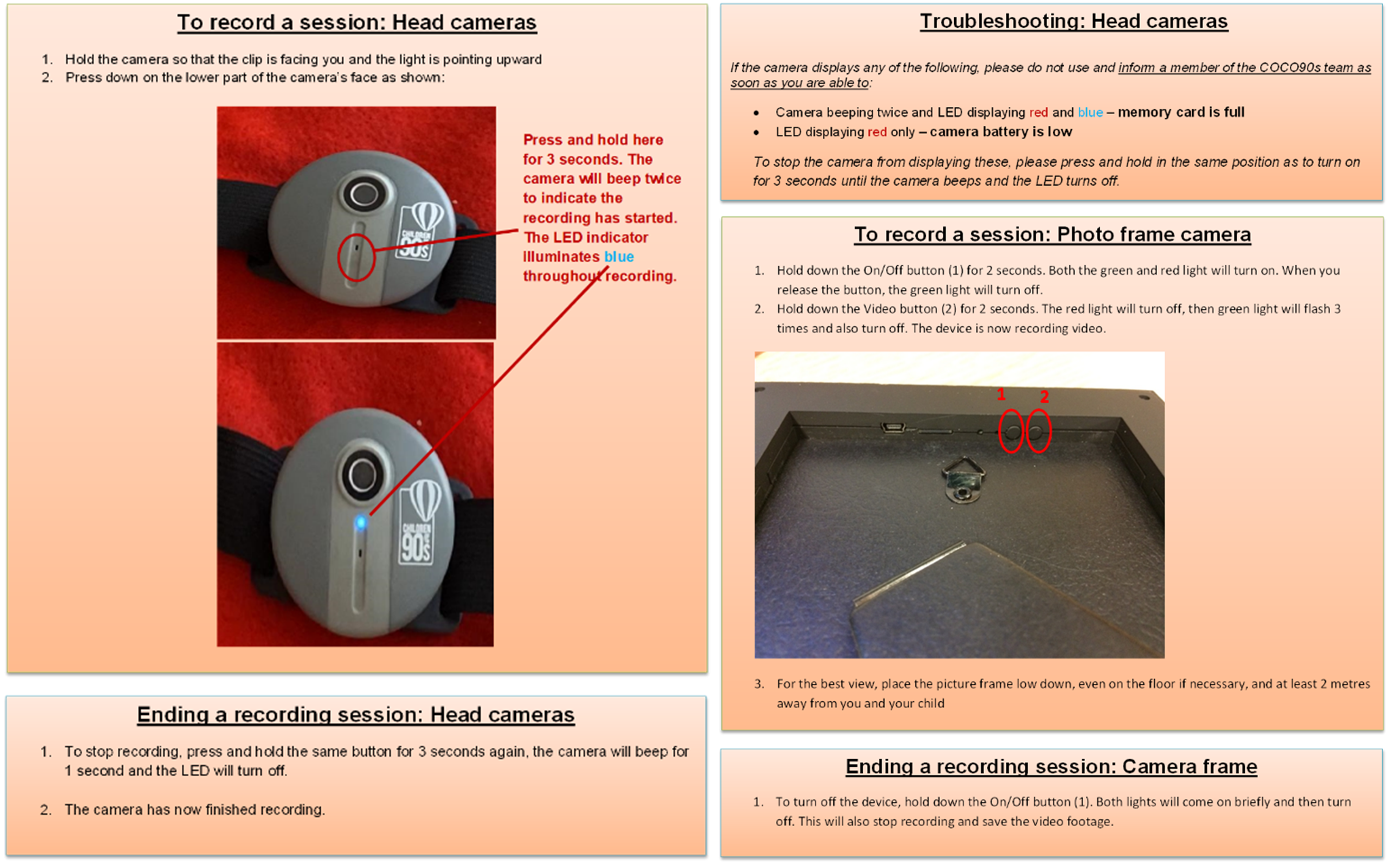


SFigure 2. This figure illustrates the most relevant coding schemes and theoretical influences on the development of the MHINT manual.


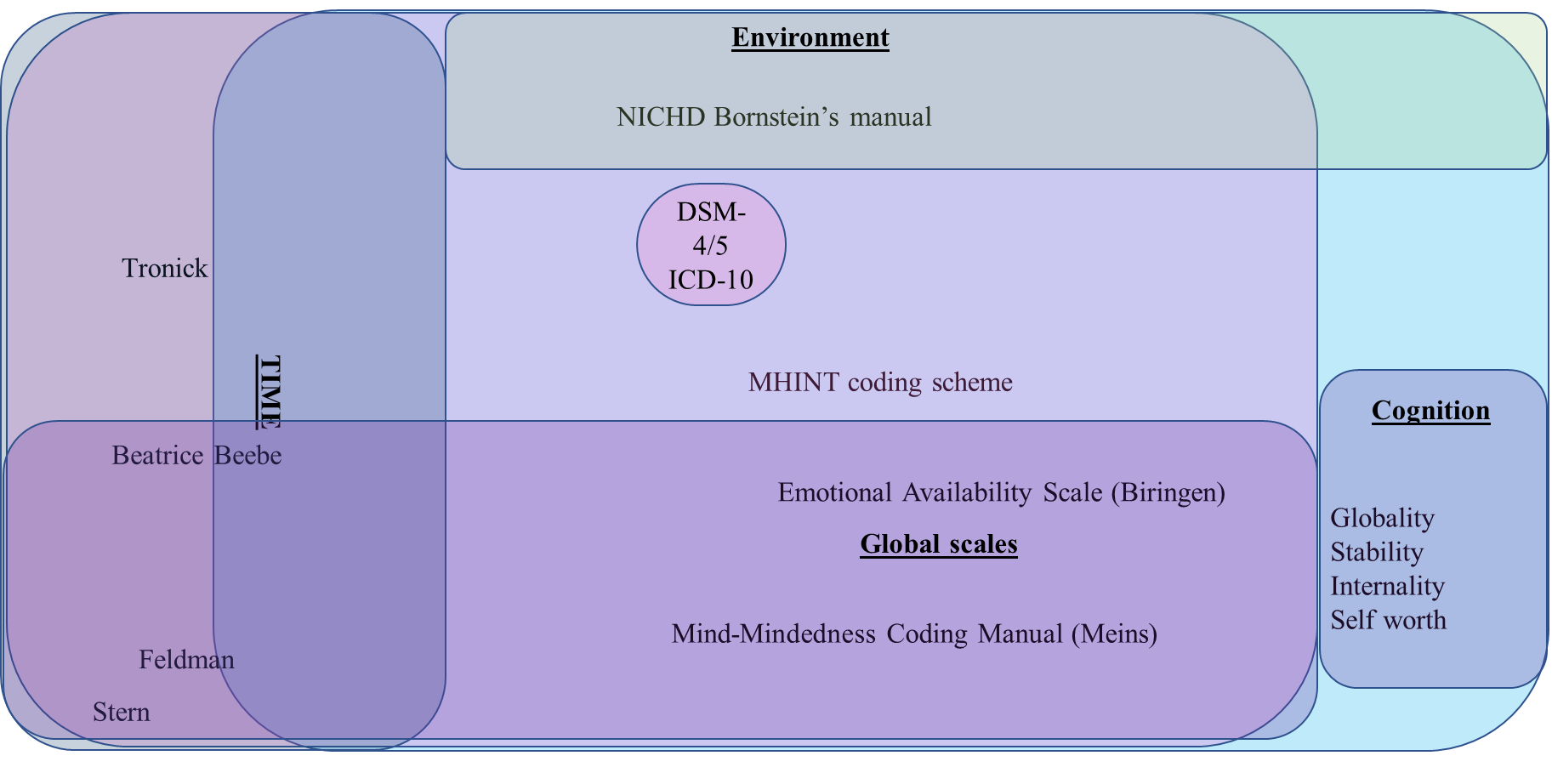


**Note**. This figure illustrates the theories and coding schemes that influenced the development and structuring of the MHINT manual and coding scheme. The MHINT manual combined multiple perspectives and theoretical influences. It built on the National Institute of Child Health and Human Development (NICHD) manual and expanded upon it by integrating codes that were both data-driven (e.g., after piloting a more basic coding scheme on ALSPAC observations, we added codes that were not present but that were considered important or useful), and theory-driven. We also integrated some codes from global scales such as the Mind-mindedness coding manual (Meins, E., & Fernyhough, 2015) and the Emotional Availability Scale (EAS) (Biringen *et al.*, 2014).

SFigure 3. Histograms of mental health measures used in this study.


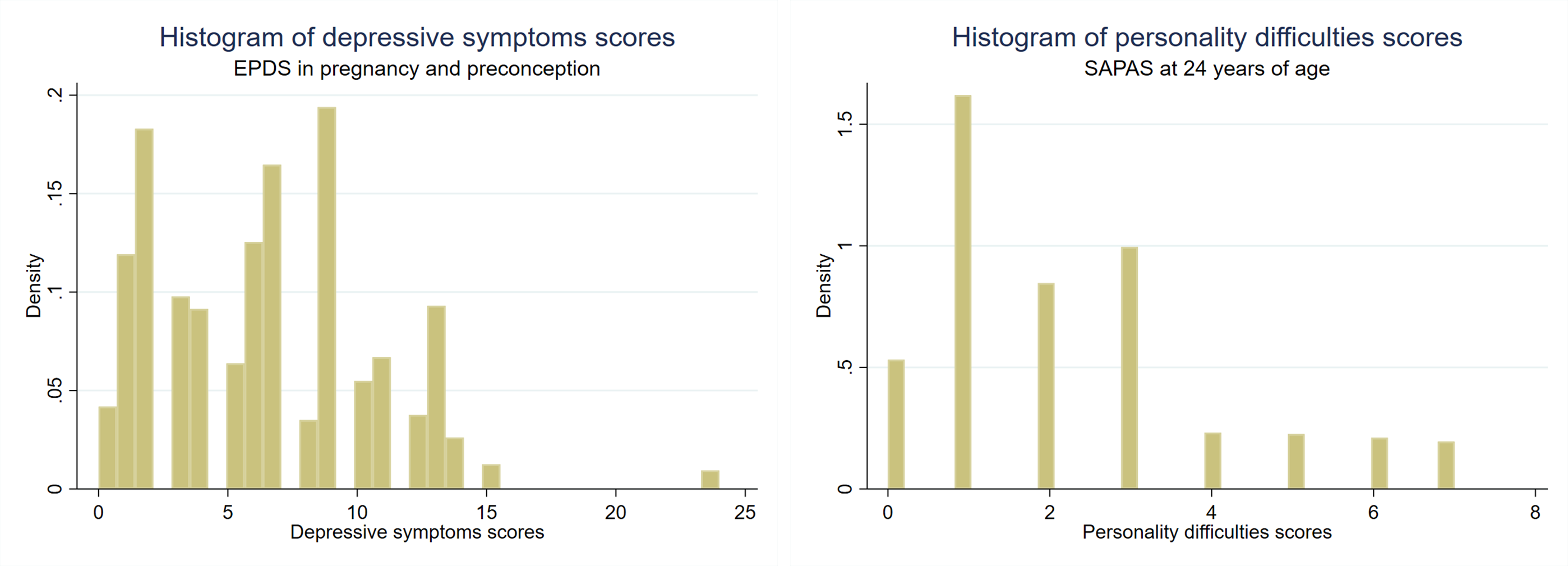


**Note.** The left panel presents a histogram of Edinburgh Postnatal Depression Scale (EPDS) scores collected during pregnancy and preconception, with depressive symptoms scores on the x-axis and density on the y-axis. The right panel presents a histogram of Standardised Assessment of Personality – Abbreviated Scale (SAPAS) scores collected at 24 years of age, with personality difficulties scores on the x-axis and density on the y-axis. The figures show the distributions of scores within the sample, indicating skewness and dispersion for each measure.

SFigure 4. Histograms of the normalised proportion of total duration score in the EPDS complete case dataset.


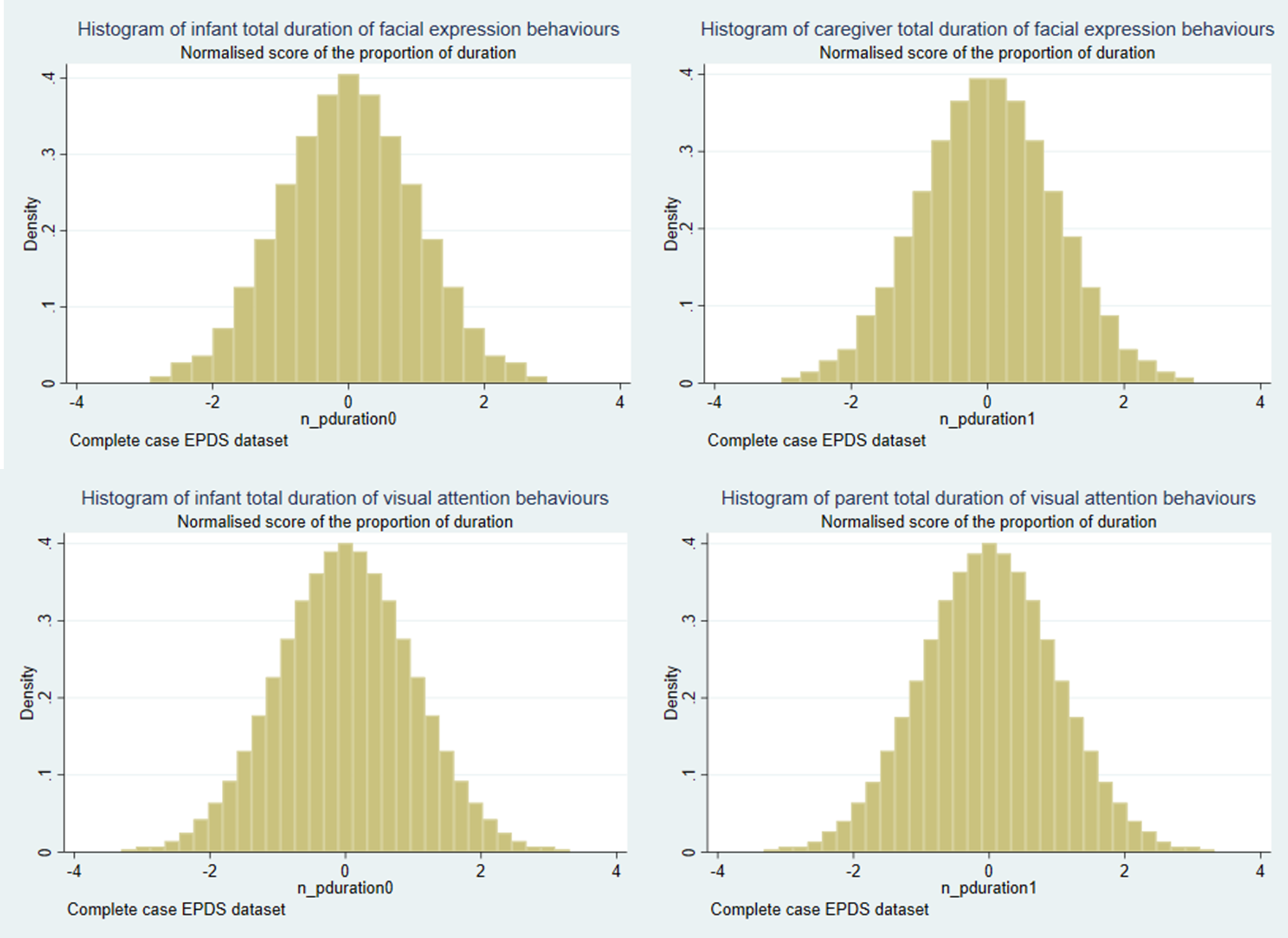


**Note.** Histograms showing the normalised score of the proportion of total duration for different behaviours in dyads where EPDS data were available. For each panel, the x-axis represents the normalised proportion of total duration, and the y-axis represents density. Distributions are approximately normal, reflecting standardisation of behaviour duration data.

SFigure 5. Histograms of the normalised proportion of total duration score in the SAPAS complete case dataset.


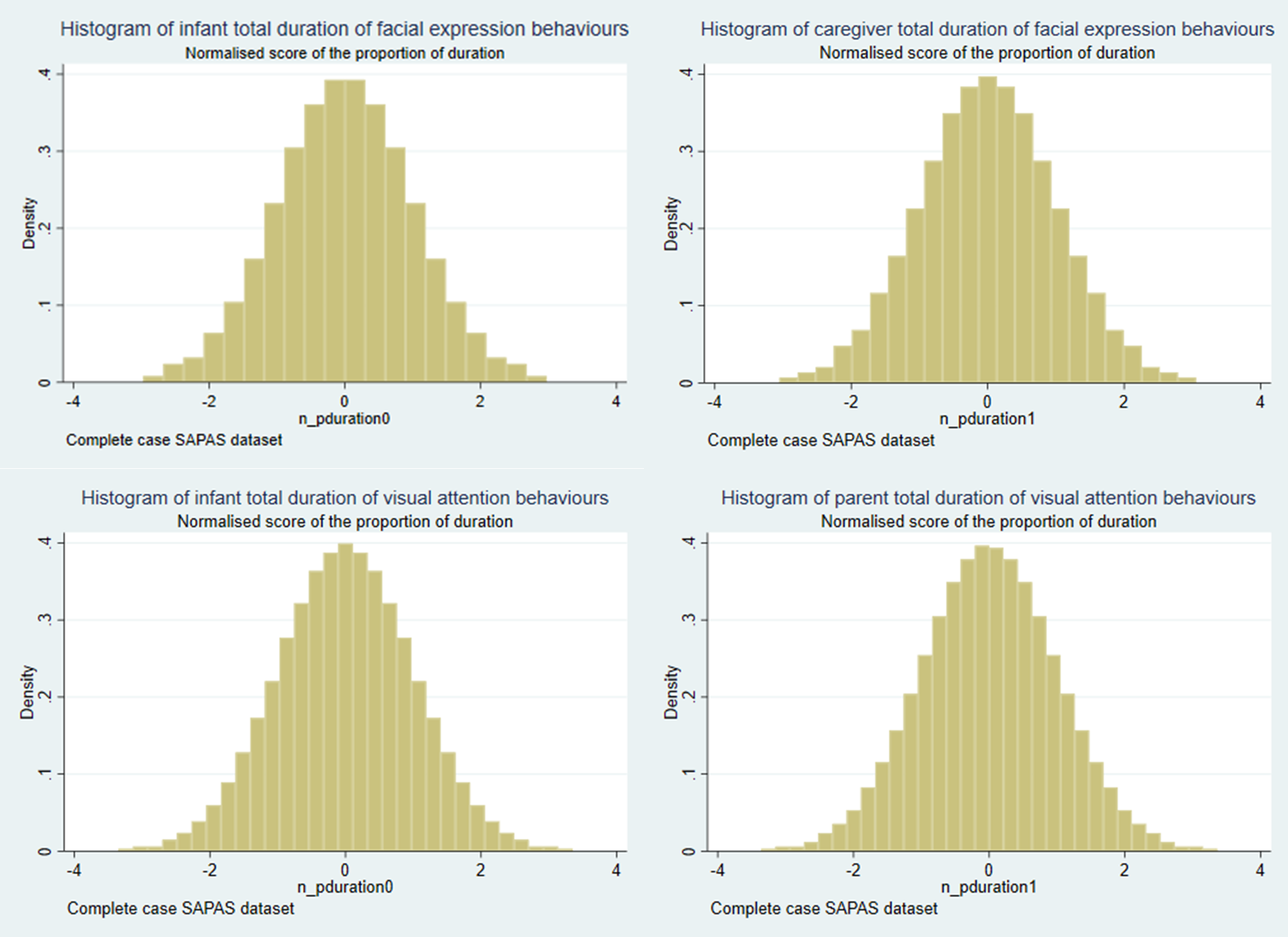


**Note.** Histograms showing the normalised score of the proportion of total duration for different behaviours in dyads where SAPAS data were available. As in SFigure 4, the x-axis represents the normalised proportion of total duration and the y-axis represents density. The distributions show symmetrical, approximately normal patterns across behaviours.

SFigure 6. Residuals from Model 4: EPDS and facial expression.


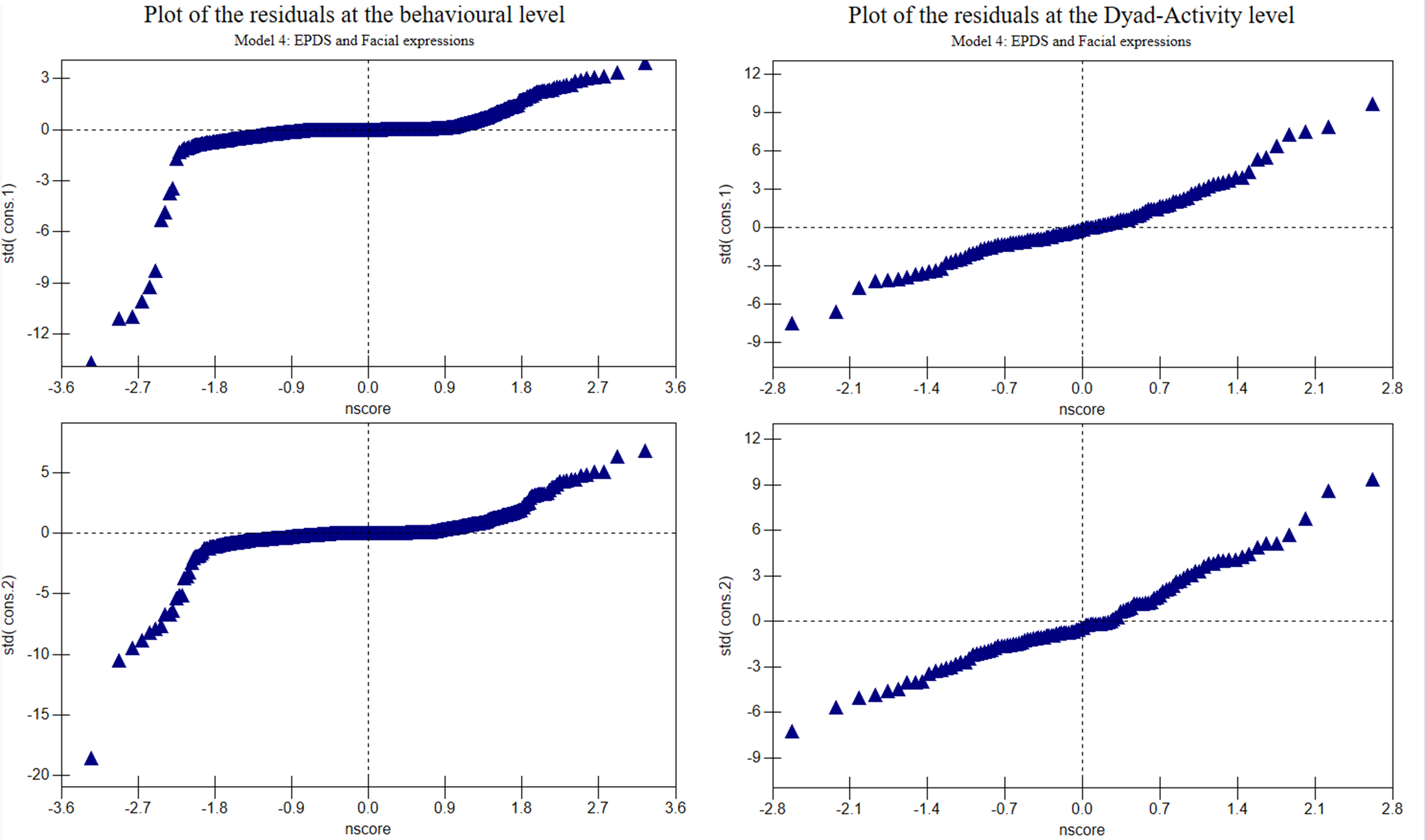


**Note.** Standardised residual plots for Model 4 examining the association between the Edinburgh Postnatal Depression Scale (EPDS) and facial expressions. The plots on the left display the residuals at the behavioural level, and the plots on the right display the residuals at the dyad-activity level. The x-axis represents the normalised score (nscore) of the proportion of total duration for each facial expression. The y-axis represents the standardised residuals of the random intercepts (std(*cons.1*) for infant facial expressions, std(*cons.2*) for parent facial expressions). Top panels correspond to the random intercepts for infants (*cons.1*), and bottom panels correspond to the random intercepts for parents (*cons.2*). Triangular markers represent individual data points. Deviation from the diagonal indicates departures from normality in the distribution of the random effects. EPDS refers to the Edinburgh Postnatal Depression Scale, a 10-item self-report measure of postnatal depressive symptoms.

SFigure 7. Residual from Model 4: EPDS and visual attention.


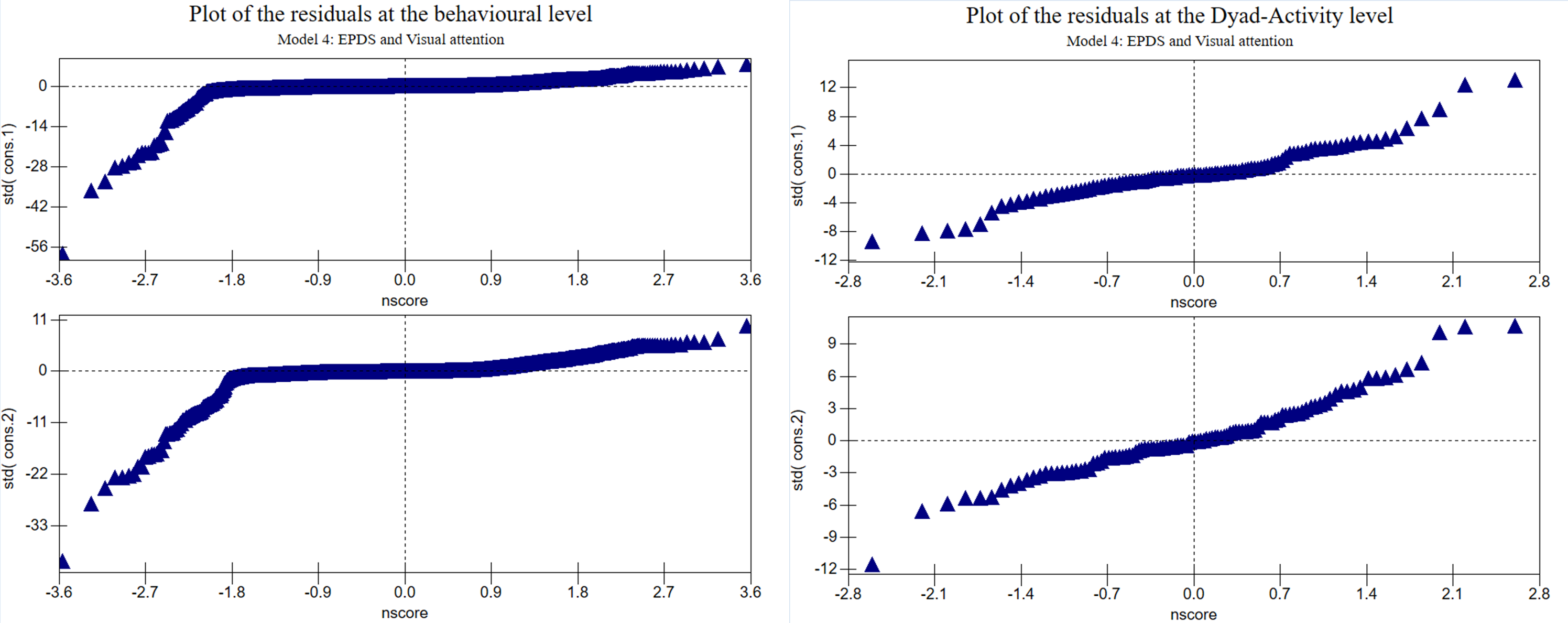


**Note.** Standardised residual plots for Model 4 examining the association between the Edinburgh Postnatal Depression Scale (EPDS) and visual attention behaviours. The plots on the left display the residuals at the behavioural level, and the plots on the right display the residuals at the dyad-activity level. The x-axis represents the normalised score (*nscore*) of the proportion of total duration for each visual attention behaviour. The y-axis represents the standardised residuals of the random intercepts (*std(cons.1)* for infant visual attention, *std(cons.2)* for parent visual attention). Top panels correspond to the random intercepts for infants (*cons.1*), and bottom panels correspond to the random intercepts for parents (*cons.2*). Triangular markers represent individual data points. Deviations from the diagonal line suggest departures from the assumption of normality in the distribution of the random effects.

SFigure 8. Residuals from Model 4: SAPAS and facial expression.


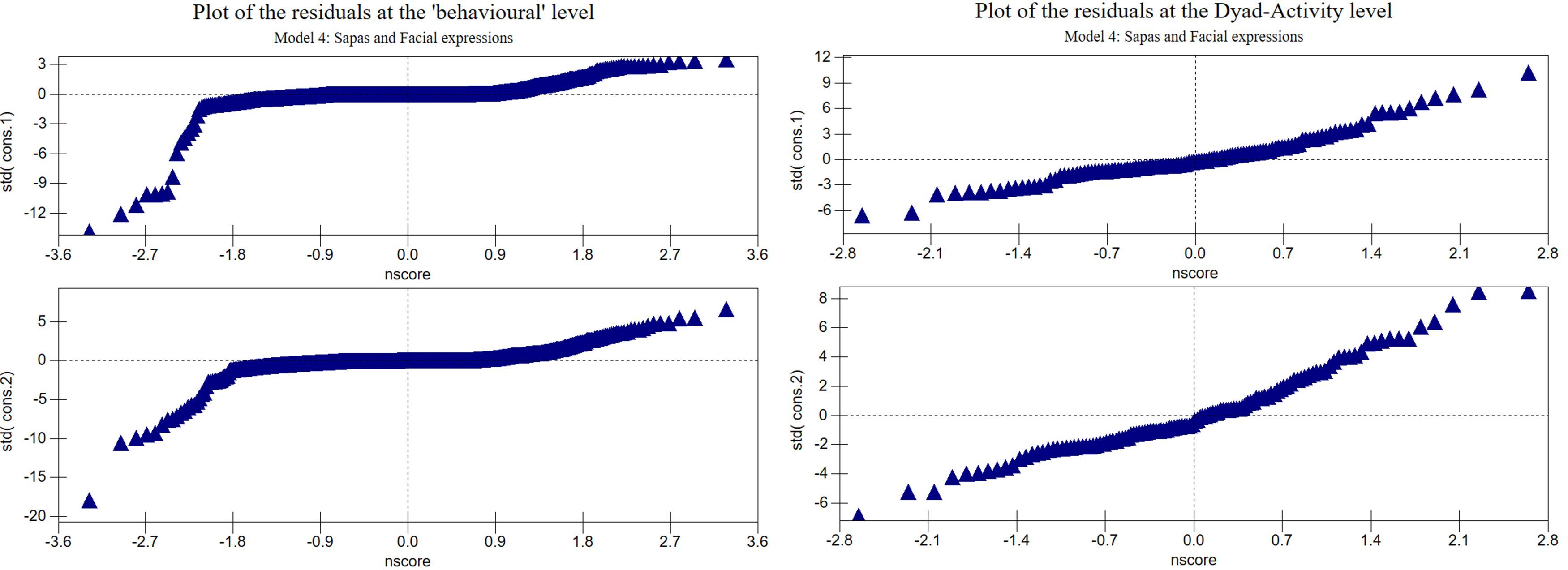


**Note.** Standardised residual plots for Model 4 examining the association between the Standardised Assessment of Personality – Abbreviated Scale (SAPAS) and facial expressions. The plots on the left display the residuals at the behavioural level, and the plots on the right display the residuals at the dyad-activity level. The x-axis represents the normalised score (*nscore*) of the proportion of total duration for each facial expression. The y-axis represents the standardised residuals of the random intercepts (*std(cons.1)* for infant facial expressions, *std(cons.2)* for parent facial expressions). Top panels correspond to the random intercepts for infants (*cons.1*), and bottom panels correspond to the random intercepts for parents (*cons.2*). Triangular markers represent individual data points. Deviations from the diagonal line suggest departures from the assumption of normality in the distribution of the random effects.

SFigure 9. Residuals from Model 4: SAPAS and visual attention


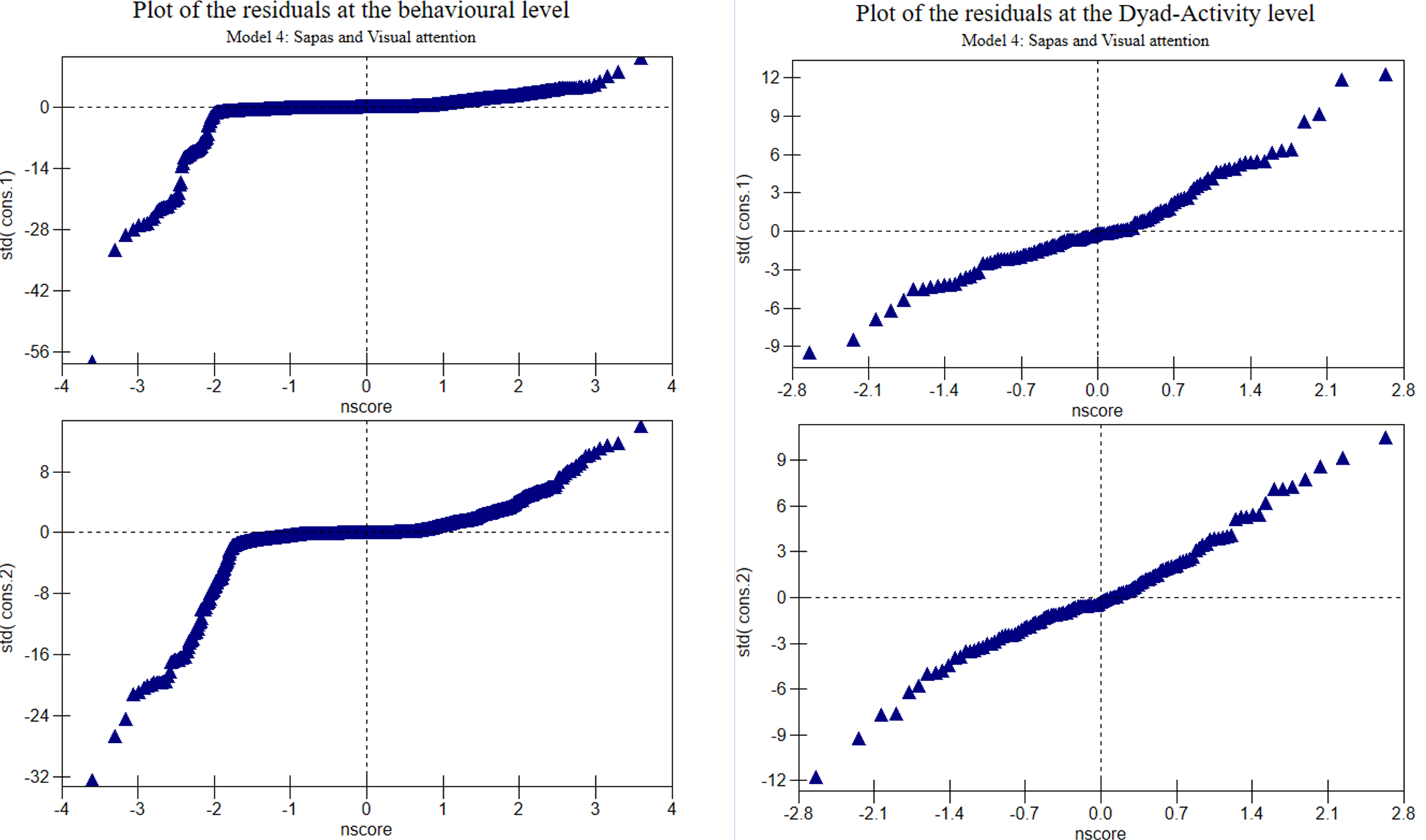


**Note.** Standardised residual plots for Model 4 examining the association between the Standardised Assessment of Personality – Abbreviated Scale (SAPAS) and visual attention behaviours. The plots on the left display the residuals at the behavioural level, and the plots on the right display the residuals at the dyad-activity level. The x-axis represents the normalised score (*nscore*) of the proportion of total duration for each visual attention behaviour. The y-axis represents the standardised residuals of the random intercepts (*std(cons.1)* for infant visual attention, *std(cons.2)* for parent visual attention). Top panels correspond to the random intercepts for infants (*cons.1*), and bottom panels correspond to the random intercepts for parents (*cons.2*). Triangular markers represent individual data points. Deviations from the diagonal line suggest departures from the assumption of normality in the distribution of the random effects.

# **Supplementary Tables**

STable 1. Descriptive statistics for total duration of infant and parent facial expressions and visual attention behaviours, comparing participants with and without missing mental health measures (EPDS and SAPAS) in 97 families.

|  | EPDS is not missing (N of participants with the EPDS=71, 73.2%)  Number of behaviours=3,435 | | | EPDS is missing (N of participants without the EPDS=26, 26.8%)  Number of behaviours=1,159 | | |
| --- | --- | --- | --- | --- | --- | --- |
| Total duration of infant behaviours using the normalised scores used in the multi-level models (see SFigure 4 for histogram) | | | | | | |
| Behaviours | **Mean** | **Std. dev.** | **Freq.** | **Mean** | **Std. dev.** | **Freq.** |
| Disgust | -0.11 | 0.58 | 13 | -0.18 | 0.83 | 7 |
| Face not visible | -0.29 | 0.58 | 69 | -0.12 | 0.42 | 27 |
| Negative | -0.18 | 0.60 | 40 | -0.07 | 0.45 | 20 |
| None of the above | -0.07 | 0.61 | 16 | -0.28 | 0.63 | 9 |
| Neutral/Alert | -0.26 | 0.59 | 85 | -0.07 | 0.41 | 28 |
| Positive | -0.18 | 0.58 | 54 | -0.07 | 0.45 | 20 |
| Smile | -0.30 | 0.76 | 40 | -0.13 | 0.47 | 15 |
| Look at caregiver 1 | 0.07 | 1.07 | 285 | 0.15 | 0.81 | 90 |
| Look at caregiver 2 | -0.17 | 0.97 | 62 | 0.04 | 0.74 | 15 |
| Look at distraction | 0.46 | 0.88 | 79 | 0.62 | 0.47 | 21 |
| Look at focus object | 0.13 | 1.09 | 313 | 0.18 | 0.81 | 108 |
| Look at object outside of view | 0.17 | 1.02 | 231 | 0.28 | 0.66 | 103 |
| Look at other object | 0.06 | 1.21 | 138 | 0.22 | 1.01 | 34 |
| Look at other person | -0.64 | 0.67 | 7 | 0.10 | 1.06 | 12 |
| Look at other child (e.g., sibling) | -0.15 | 1.07 | 51 | 0.20 | 0.27 | 21 |
| No visual attention | 0.49 | 0.92 | 30 | 0.18 | 1.04 | 17 |
| Not possible to code visual attention | 0.13 | 0.80 | 87 | 0.29 | 0.60 | 27 |
| Total | 0.04 | 0.99 | 1,600 | 0.15 | 0.73 | 574 |
| Total duration of parent behaviours using the normalised scores used in the multi-level models (see SFigure 4 for histogram) | | | | | | |
| Behaviours | **Mean** | **Std. dev.** | **Freq.** | **Mean** | **Std. dev.** | **Freq.** |
| Disgust | -0.87 | 0.36 | 4 | -0.22 | 0.48 | 5 |
| Face not visible | -0.37 | 0.55 | 103 | -0.32 | 0.40 | 27 |
| None of the above | -0.29 | 0.69 | 32 | -0.30 | 0.69 | 11 |
| Positive | -0.32 | 0.60 | 85 | -0.35 | 0.53 | 24 |
| Smile | -0.31 | 0.59 | 83 | -0.47 | 0.70 | 23 |
| Mock surprise | -0.25 | 0.60 | 53 | -0.04 | 0.49 | 10 |
| Negative | -0.61 | 0.63 | 18 | -0.5 | 0.62 | 4 |
| Neutral/Alert | -0.30 | 0.51 | 95 | -0.34 | 0.50 | 25 |
| Surprise | -0.00 | 0.19 | 2 | -1.39 | 0 | 1 |
| ‘Woe’ face | -0.84 | 1.17 | 6 | -0.95 | 1.32 | 2 |
| Look at caregiver 1 | 0.38 | 1.36 | 23 | -0.52 | 0.47 | 6 |
| Look at caregiver 2 | -0.57 | 1.51 | 35 | -0.78 | 0.52 | 7 |
| Look at distraction | 0.14 | 1.21 | 92 | -0.17 | 1.11 | 40 |
| Look at focus object | 0.09 | 1.16 | 322 | -0.07 | 1.21 | 105 |
| Look at infant | 0.09 | 1.12 | 316 | 0.14 | 1.25 | 97 |
| Look at object outside of view | 0.02 | 1.12 | 223 | -0.05 | 1.16 | 82 |
| Look at other object | 0.02 | 1.17 | 184 | 0.14 | 1.32 | 60 |
| Look at other person | -0.37 | 1.01 | 6 | -0.09 | 1.15 | 10 |
| Look at other child (e.g., sibling) | 0.12 | 0.93 | 55 | 1.26 | 1.30 | 17 |
| No visual attention | -0.54 | 0.87 | 12 | -0.49 | 1.08 | 7 |
| Not possible to code visual attention | 0.12 | 0.93 | 86 | 0.26 | 0.88 | 22 |
| Total | -0.05 | 1.04 | 1,835 | -0.05 | 1.11 | 585 |
|  | SAPAS was not missing (N of participants with the SAPAS=83, 85.6%)  Number of behaviours available=4,090 | | | SAPAS was missing (N of participants without the SAPAS=14, 14.4%)  Number of behaviours available=504 | | |
| Total duration of infant behaviours using the normalised scores used in the multi-level models (see SFigure 5 for histogram) | | | | | | |
| Behaviours | **Mean** | **Std. dev.** | **Freq.** | **Mean** | **Std. dev.** | **Freq.** |
| Disgust | -0.09 | 0.63 | 19 | -1.06 | - | 1 |
| Face not visible | -0.23 | 0.56 | 81 | -0.34 | 0.44 | 15 |
| Negative | -0.08 | 0.51 | 51 | -0.48 | 0.72 | 9 |
| Neutral/Alert | -0.19 | 0.54 | 99 | -0.40 | 0.66 | 14 |
| None of the above | -0.13 | 0.64 | 21 | -0.25 | 0.52 | 4 |
| Positive | -0.13 | 0.51 | 66 | -0.39 | 0.80 | 8 |
| Smile | -0.21 | 0.68 | 48 | -0.54 | 0.80 | 7 |
| Look at caregiver 1 | 0.08 | 0.98 | 334 | 0.17 | 1.25 | 41 |
| Look at caregiver 2 | -0.19 | 0.78 | 64 | 0.16 | 1.47 | 13 |
| Look at distraction | 0.51 | 0.85 | 91 | 0.28 | 0.12 | 9 |
| Look at focus object | 0.12 | 1.02 | 379 | 0.31 | 1.10 | 42 |
| Look at object outside of view | 0.19 | 0.89 | 299 | 0.27 | 1.17 | 35 |
| Look at other object | 0.10 | 1.13 | 158 | -0.09 | 1.66 | 14 |
| Look at other person | -0.25 | 1.08 | 15 | 0.11 | 0.52 | 4 |
| Look at other child (e.g., sibling) | 0.01 | 0.92 | 67 | -0.83 | 0.48 | 5 |
| No visual attention | 0.37 | 0.86 | 41 | 0.43 | 1.61 | 6 |
| Not possible to code visual attention | 0.18 | 0.76 | 102 | 0.08 | 0.75 | 12 |
| Total | 0.08 | 0.91 | 1,935 | 0.04 | 1.10 | 239 |
| Total duration of parent behaviours using the normalised scores used in the multi-level models (see SFigure 5 for histogram) | | | | | | |
| Behaviours | **Mean** | **Std. dev.** | **Freq.** | **Mean** | **Std. dev.** | **Freq.** |
| Disgust | -0.44 | 0.59 | 7 | -0.74 | 0.21 | 2 |
| Face not visible | -0.33 | 0.50 | 116 | -0.62 | 0.66 | 14 |
| Mock surprise | -0.21 | 0.59 | 58 | -0.24 | 0.69 | 5 |
| Negative | -0.61 | 0.65 | 19 | -0.49 | 0.32 | 3 |
| Neutral/Alert | -0.27 | 0.47 | 107 | -0.65 | 0.67 | 13 |
| None of the above | -0.30 | 0.69 | 38 | -0.24 | 0.73 | 5 |
| Positive | -0.28 | 0.56 | 96 | -0.64 | 0.67 | 13 |
| Smile | -0.31 | 0.59 | 95 | -0.65 | 0.75 | 11 |
| Surprise | -0.47 | 0.81 | 3 | - | - | - |
| ‘Woe’ face | -0.72 | 1.11 | 7 | -1.88 | - | 1 |
| Look at caregiver 1 | 0.07 | 1.25 | 25 | 0.99 | 1.28 | 4 |
| Look at caregiver 2 | -0.51 | 1.42 | 38 | -1.49 | 0.85 | 4 |
| Look at distraction | 0.07 | 1.20 | 113 | -0.10 | 1.14 | 19 |
| Look at focus object | 0.09 | 1.17 | 377 | -0.28 | 1.20 | 50 |
| Look at infant | 0.14 | 1.13 | 367 | -0.22 | 1.26 | 46 |
| Look at object outside of view | 0.02 | 1.14 | 279 | -0.26 | 1.00 | 26 |
| Look at other object | 0.09 | 1.20 | 224 | -0.40 | 1.25 | 20 |
| Look at other person | 0.23 | 1.01 | 11 | -1.13 | 0.37 | 5 |
| Look at other child (e.g., sibling) | 0.67 | 0.96 | 61 | -1.18 | 0.64 | 11 |
| No visual attention | -0.51 | 0.95 | 18 | -0.72 | - | 1 |
| Not possible to code visual attention | 0.15 | 0.91 | 96 | 0.15 | 0.98 | 12 |
| Total | -0.01 | 1.05 | 2,155 | -0.38 | 1.07 | 265 |

## Descriptive and random effects of the models

STable 2. Adjusted model of the EPDS on visual attention behaviours in infants (first part of the equation) and parents (second part of the equation).

| Fixed Part | Adjusted model | 95% CrIs |
| --- | --- | --- |
| cons.1 | 0.90 | -1.43, 4.11 |
| Look at caregiver 1 | -0.05 | -0.11, 0.02 |
| Look at caregiver 2 | -0.09 | -0.21, 0.02 |
| Look at distraction | -0.10 | -0.20, 0.01 |
| Look at object outside of view | -0.04 | -0.10, 0.03 |
| Look at other object | -0.08 | -0.16, 0.00 |
| Look at other person | 0.38 | -0.25, 1.01 |
| Look at sibling | -0.10 | -0.23, 0.03 |
| No visual attention | 0.13 | -0.02, 0.26 |
| Not possible to code visual attention | -0.02 | -0.12, 0.07 |
| cons.2 | -0.34 | -2.34, 1.79 |
| Look at caregiver 2 | -0.28 | -0.44, -0.12 |
| Look at distraction | -0.14 | -0.24, -0.03 |
| Look at Infant | 0.07 | 0.00, 0.13 |
| Look at object outside of view | 0.00 | -0.07, 0.07 |
| Look at other object | 0.00 | -0.08, 0.07 |
| Look at other person | 0.04 | -0.52, 0.59 |
| Look at sibling | 0.06 | -0.08, 0.20 |
| No visual attention | -0.63 | -0.88, -0.40 |
| Not possible to code visual attention | 0.01 | -0.09, 0.11 |

**Note.** EPDS = Edinburgh Postnatal Depression Scale; *cons.1* = intercept for infant behaviours; *cons.2* = intercept for parent behaviours; 95% CrIs = 95% Credible Intervals.
Beta coefficients represent the association between EPDS scores and the normalised proportion of total duration of each visual attention behaviour. Negative coefficients indicate a lower proportion of time spent in the behaviour per 1-standard deviation (SD) increase in EPDS score, and positive coefficients indicate a higher proportion.

STable 3. Adjusted model of the EPDS on total duration of facial expressions in infants (first part of the equation) and parents (second part of the equation).

|  | Model4 | 95% CrIs |
| --- | --- | --- |
| cons.1 | -0.65 | -2.83, 1.19 |
| Disgust | -0.22 | -0.44, 0.00 |
| Negative | -0.14 | -0.29, 0.01 |
| Neutral/Alert | 0.03 | -0.08, 0.14 |
| None of the above | 0.08 | -0.13, 0.30 |
| Positive | 0.01 | -0.12, 0.15 |
| Smile | -0.07 | -0.22, 0.07 |
| cons.2 | -1.18 | -3.14, 0.74 |
| Disgust | -1.28 | -1.72, -0.84 |
| Mock surprise | -0.16 | -0.30, -0.01 |
| Negative | -0.76 | -1.01, -0.51 |
| Neutral/Alert | 0.03 | -0.09, 0.15 |
| None of the above | -0.14 | -0.32, 0.04 |
| Positive | 0.05 | -0.08, 0.17 |
| Smile | 0.05 | -0.07, 0.18 |
| Surprise | 0.11 | -2.12, 2.33 |
| ‘Woe’ Face | -1.35 | -1.78, -0.92 |

**Note.** This table presents results from an adjusted model examining the association between parental depressive symptoms, measured using the Edinburgh Postnatal Depression Scale (EPDS), and the total duration of facial expressions displayed by infants (first part of the equation) and parents (second part of the equation). CrI refers to the 95% Credible Interval, indicating the range within which the parameter is likely to fall with 95% probability. "*cons.1*" and "*cons.2*" represent the intercept terms for the infant and parent models, respectively. Negative coefficients indicate an association between higher EPDS scores and shorter total durations of the corresponding facial expression, whereas positive coefficients indicate an association with longer total durations. Credible intervals that do not include zero provide stronger evidence for an association.

STable 4. Adjusted model of SAPAS on total duration of visual attention in infants (first part of the equation) and parents (second part of the equation).

|  | Model 4 | 95% CrIs |
| --- | --- | --- |
| cons.1 | 0.38 | -1.43, 2.02 |
| Look at caregiver 1 | -0.05 | -0.10, 0.02 |
| Look at caregiver 2 | -0.09 | -0.21, 0.02 |
| Look at distraction | -0.07 | -0.17, 0.04 |
| Look at object outside of view | -0.01 | -0.08, 0.05 |
| Look at other object | -0.02 | -0.10, 0.06 |
| Look at other person | -0.12 | -0.36, 0.12 |
| Look at sibling | -0.03 | -0.15, 0.10 |
| No visual attention | 0.08 | -0.06, 0.22 |
| Not possible to code visual attention | 0.08 | -0.02, 0.17 |
| cons.2 | -0.07 | -2.01, 1.77 |
| Look at caregiver 2 | -0.22 | -0.40, -0.06 |
| Look at distraction | -0.25 | -0.36, -0.14 |
| Look at Infant | 0.08 | 0.01, 0.14 |
| Look at object outside of view | -0.04 | -0.11, 0.04 |
| Look at other object | 0.02 | -0.06, 0.10 |
| Look at other person | 0.04 | -0.28, 0.35 |
| Look at sibling | 0.25 | 0.10, 0.40 |
| No visual attention | -0.46 | -0.69, -0.22 |
| Not possible to code visual attention | -0.03 | -0.14, 0.08 |

**Note.** This table presents results from an adjusted model examining the association between personality traits, measured using the Standardised Assessment of Personality – Abbreviated Scale (SAPAS), and the total duration of visual attention behaviours displayed by infants (first part of the equation) and parents (second part of the equation). CrI refers to the 95% Credible Interval indicating the range within which the parameter is likely to fall with 95% probability. "*cons.1*" and "*cons.2*" represent the intercept terms for the infant and parent models, respectively. Negative coefficients indicate an association between higher SAPAS scores and shorter total durations of the corresponding visual attention behaviour, whereas positive coefficients indicate an association with longer total durations. Credible intervals that do not include zero provide stronger evidence for an association.

STable 5. Adjusted model of SAPAS on total duration of facial expression in infants (first part of the equation) and parents (second part of the equation).

| Adjusted model 4 (SAPAS and facial expressions) | | |
| --- | --- | --- |
|  | **Beta** | **95% CrI** |
| cons.1 | -0.59 | -2.90, 1.72 |
| Disgust | -0.28 | -0.51, -0.05 |
| Negative | -0.18 | -0.33, -0.03 |
| Neutral/Alert | 0.01 | -0.11, 0.13 |
| None of the above | 0.00 | -0.21, 0.21 |
| Positive | 0.01 | -0.13, 0.14 |
| Smile | -0.14 | -0.29, 0.02 |
| cons.2 | -0.65 | -2.80, 1.47 |
| Disgust | -0.95 | -1.30, -0.59 |
| Mock surprise | -0.13 | -0.27, 0.02 |
| Negative | -0.80 | -1.04, -0.56 |
| Neutral/Alert | 0.03 | -0.09, 0.15 |
| None of the above | -0.19 | -0.36, 0.00 |
| Positive | 0.04 | -0.09, 0.16 |
| Smile | 0.01 | -0.12, 0.13 |
| Surprise | -0.45 | -1.17, 0.27 |
| ‘Woe’ face | -1.08 | -1.46, -0.70 |

**Note.** This table presents results from an adjusted model examining the association between personality traits, measured using the Standardised Assessment of Personality – Abbreviated Scale (SAPAS), and the total duration of facial expressions displayed by infants (first part of the equation) and parents (second part of the equation). CrI refers to the 95% Credible Interval indicating the range within which the parameter is likely to fall with 95% probability. "*cons.1*" and "*cons.2*" represent the intercept terms for the infant and parent models, respectively. Negative coefficients indicate an association between higher SAPAS scores and shorter total durations of the corresponding facial expression, whereas positive coefficients indicate an association with longer total durations. Credible intervals that do not include zero provide stronger evidence for an association.

STable 6. Random effects component of all the models presented.

| Random part of model 4 (EPDS and visual attention) | | | |
| --- | --- | --- | --- |
| Level: dyad_activity_long | **Beta** | **Cred Int 2.5%** | **Cred Int 97.5%** |
| Var(cons.1) | 0.78 | 0.59 | 1.04 |
| Covar(cons.2/cons.1) | 0.22 | 0.06 | 0.40 |
| Corr(cons.2/cons.1) | 0.28 | 0.08 | 0.46 |
| Var(cons.2) | 0.80 | 0.61 | 1.06 |
| Level: ID_long |  |  |  |
| Var(cons.1) | 0.14 | 0.13 | 0.16 |
| Covar(cons.2/cons.1) | 0.05 | -0.08 | 0.12 |
| Corr(cons.2/cons.1) | 0.34 | -0.53 | 0.79 |
| Var(cons.2) | 0.16 | 0.15 | 0.18 |
| Random part of model 4 (EPDS and facial expression) | | | |
| Level: dyad_activity_long | **Beta** | **Cred Int 2.5%** | **Cred Int 97.5%** |
| Var(cons.1) | 0.81 | 0.60 | 1.08 |
| Covar(cons.2/cons.1) | 0.55 | 0.38 | 0.77 |
| Corr(cons.2/cons.1) | 0.70 | 0.58 | 0.80 |
| Var(cons.2) | 0.76 | 0.57 | 1.01 |
| Level: ID_long |  |  |  |
| Var(cons.1) | 0.14 | 0.12 | 0.17 |
| Covar(cons.2/cons.1) | 0.01 | -0.12 | 0.12 |
| Corr(cons.2/cons.1) | 0.07 | -0.75 | 0.75 |
| Var(cons.2) | 0.18 | 0.16 | 0.21 |
| Random part of model 4 (SAPAS and visual attention) | | | |
| Level: dyad_activity_long | **Beta** | **Cred Int 2.5%** | **Cred Int 97.5%** |
| Var(cons.1) | 0.80 | 0.60 | 1.06 |
| Covar(cons.2/cons.1) | 0.13 | -0.02 | 0.29 |
| Corr(cons.2/cons.1) | 0.17 | -0.02 | 0.36 |
| Var(cons.2) | 0.74 | 0.57 | 0.97 |
| Level: ID_long |  |  |  |
| Var(cons.1) | 0.17 | 0.16 | 0.18 |
| Covar(cons.2/cons.1) | 0.06 | -0.08 | 0.15 |
| Corr(cons.2/cons.1) | 0.28 | -0.44 | 0.79 |
| Var(cons.2) | 0.22 | 0.21 | 0.24 |
| Random part of model 4 (SAPAS and facial expression) | | | |
| Level: dyad_activity_long | **Beta** | **Cred Int 2.5%** | **Cred Int 97.5%** |
| Var(cons.1) | 0.84 | 0.63 | 1.11 |
| Covar(cons.2/cons.1) | 0.55 | 0.38 | 0.75 |
| Corr(cons.2/cons.1) | 0.69 | 0.56 | 0.79 |
| Var(cons.2) | 0.75 | 0.56 | 0.98 |
| Level: ID_long |  |  |  |
| Var(cons.1) | 0.18 | 0.15 | 0.21 |
| Covar(cons.2/cons.1) | 0.00 | -0.14 | 0.15 |
| Corr(cons.2/cons.1) | -0.02 | -0.73 | 0.77 |
| Var(cons.2) | 0.20 | 0.18 | 0.23 |

**Note.** This table reports the random effects component of the final adjusted models (Model 4) examining associations between mental health measures—the Edinburgh Postnatal Depression Scale (EPDS) and the Standardised Assessment of Personality – Abbreviated Scale (SAPAS)—and observed infant and parent behaviours (facial expressions and visual attention). Estimates are shown for both hierarchical levels in the model: **dyad_activity_long**, which accounts for shared variance at the level of specific dyadic interaction episodes, and **ID_long**, which accounts for variance between individuals across sessions. "Var(*cons.1*)" and "Var(*cons.2*)" refer to the variances of the random intercepts for infants and parents, respectively. "Covar(*cons.2/cons.1*)" and "Corr(*cons.2/cons.1*)" represent the covariance and correlation, respectively, between infant and parent random intercepts within the same level. Estimates are presented alongside their 95% Credible Intervals (CrIs), which indicate the range within which the parameter is likely to fall with 95% probability.

STable 7. Model fit diagnostics for all included models.

| Models | Description | DIC | pD |
| --- | --- | --- | --- |
| Personality difficulties (SAPAS) and facial expressions | |  |  |
| Model 0 | Bivariate two-level model with constants only | 3211.50 | 891.32 |
| Model 1 | Bivariate two-level model with constants and behaviours | 3088.32 | 964.08 |
| Model 2 | Bivariate two-level model with constants, behaviours, and SAPAS | 2990.69 | 915.20 |
| Model 3 | Bivariate two-level model with constants, behaviours, SAPAS, and interactions between SAPAS and the included behaviours | 2991.44 | 949.32 |
| Model 4 | Bivariate two-level model with constants, behaviours, SAPAS, interactions between SAPAS and the included behaviours, and basic covariates | 2960.85 | 936.082 |
| Models | **Description** | **DIC** | **pD** |
| Personality difficulties (SAPAS) and visual attention | |  |  |
| Model 0 | Bivariate two-level model with constants only | 9755.58 | 2704.16 |
| Model 1 | Bivariate two-level model with constants and behaviours | 9084.83 | 2455.72 |
| Model 2 | Bivariate two-level model with constants, behaviours, and SAPAS | 9573.66 | 2957.22 |
| Model 3 | Bivariate two-level model with constants, behaviours, SAPAS, and interactions between SAPAS and the included behaviours | 8913.90 | 2501.27 |
| Model 4 | Bivariate two-level model with constants, behaviours, SAPAS, interactions between SAPAS and the included behaviours, and basic covariates | 9601.63 | 2877.42 |
| Models | **Description** | **DIC** | **pD** |
| Depressive symptoms (EPDS) and facial expressions | |  |  |
| Model 0 | Bivariate two-level model with constants only | 2634.81 | 856.341 |
| Model 1 | Bivariate two-level model with constants and behaviours | 2343.89 | 825.103 |
| Model 2 | Bivariate two-level model with constants, behaviours, and EPDS | 2417.82 | 895.01 |
| Model 3 | Bivariate two-level model with constants, behaviours, EPDS, and interactions between EPDS and the included behaviours | 2445.75 | 918.30 |
| Model 4 | Bivariate two-level model with constants, behaviours, EPDS, interactions between EPDS and the included behaviours, and basic covariates | 2313.61 | 840.22 |
| Models | **Description** | **DIC** | **pD** |
| Depressive symptoms (EPDS) and visual attention | |  |  |
| Model 0 | Bivariate two-level model with constants only | 7187.76 | 2355.45 |
| Model 1 | Bivariate two-level model with constants and behaviours | 6553.87 | 2111.09 |
| Model 2 | Bivariate two-level model with constants, behaviours, and EPDS | 6871.34 | 2289.20 |
| Model 3 | Bivariate two-level model with constants, behaviours, EPDS, and interactions between EPDS and the included behaviours | 6174.14 | 1982.85 |
| Model 4 | Bivariate two-level model with constants, behaviours, EPDS, interactions between EPDS and the included behaviours, and basic covariates | 6618.95 | 2360.51 |

**Note.** In general, pD (difference between the posterior mean of the deviance and the deviance at the posterior means of the parameters of interest) is the effective number of parameters, and thus represents a measure of model complexity(Spiegelhalter *et al.*, 2002; Leckie and Charlton, 2013). Deviance Information Criterion (DIC) is a Bayesian method for model comparison. DIC is given by the sum of the posterior mean of the deviance and the pD. Smaller values of the DIC indicate a better model fit that is the model that would best predict a replicate dataset which has the same structure as that currently observed. DIC appropriately takes into account model complexity(Spiegelhalter *et al.*, 2002).

STable 8. Adjusted model 5 models of facial expressions with the EPDS and the SAPAS in complete case analyses (participants with both the EPDS and the SAPAS).

| Exploring patterns of visual attention in dyads where the parent completed a measure of EPDS or SAPAS (in those who completed both the EPDS and the SAPAS) | | | | |
| --- | --- | --- | --- | --- |
| Interaction with EPDS or SAPAS score and each of the behaviours | Beta  (Adjusted estimates) EPDS | 95% CrIs | Beta  (Adjusted estimates) SAPAS | 95% CrIs |
| Infant visual attention |  |  |  |  |
| Look at Caregiver1 | 0.06 | -0.01, 0.13 | 0.06 | -0.01, 0.12 |
| Look at Caregiver2 | 0.03 | -0.10, 0.15 | -0.02 | -0.13, 0.09 |
| Look at Distraction | 0.09 | -0.01, 0.18 | 0.09 | -0.03, 0.20 |
| Look at Object Outside of View | 0.08 | 0.01, 0.15 | 0.01 | -0.06, 0.08 |
| Look at other Object | 0.00 | -0.09, 0.09 | 0.08 | -0.01, 0.16 |
| Look at other Person | -0.13 | -0.65, 0.39 | 0.28 | -0.05, 0.61 |
| Look at other Child (e.g., sibling) | 0.31 | 0.17, 0.44 | 0.15 | 0.06, 0.25 |
| No Visual Attention | -0.03 | -0.19, 0.13 | -0.01 | -0.18, 0.16 |
| Not Possible to Code | 0.03 | -0.08, 0.14 | 0.02 | -0.09, 0.13 |
| Caregiver visual attention |  |  |  |  |
| Look at Caregiver 2 | 0.40 | 0.21, 0.61 | 0.02 | -0.11, 0.15 |
| Look at Distraction | 0.06 | -0.06, 0.17 | -0.02 | -0.14, 0.10 |
| Look at Infant | 0.03 | -0.04, 0.09 | -0.01 | -0.09, 0.05 |
| Look at Object Outside of View | 0.03 | -0.03, 0.10 | -0.04 | -0.12, 0.04 |
| Look at other Object | -0.03 | -0.11, 0.04 | -0.03 | -0.11, 0.05 |
| Look at other Person | 0.04 | -0.43, 0.53 | 0.02 | -0.54, 0.59 |
| Look at other child (e.g., sibling) | -0.03 | -0.20, 0.14 | -0.02 | -0.12, 0.09 |
| No Visual Attention | 0.20 | -0.10, 0.48 | 0.23 | -0.03, 0.48 |
| Not Possible to Code | 0.05 | -0.06, 0.15 | 0.00 | -0.11, 0.11 |

STable 9. Adjusted model 5 models of facial expressions with the EPDS and the SAPAS in complete case analyses (participants with both the EPDS and the SAPAS).

| Exploring patterns of facial expressions in dyads where the parent completed a measure of EPDS or SAPAS (in those who completed both the EPDS and the SAPAS) | | | | |
| --- | --- | --- | --- | --- |
| Interaction with EPDS or SAPAS score and each of the behaviours | Beta  (Adjusted estimates) EPDS | 95% CrIs | Beta  (Adjusted estimates) SAPAS | 95% CrIs |
| Infant facial expressions |  |  |  |  |
| Disgust | -0.12 | -0.37, 0.12 | -0.23 | -0.45, -0.01 |
| Negative | -0.11 | -0.29, 0.08 | -0.11 | -0.29, 0.07 |
| Neutral | -0.02 | -0.14, 0.11 | -0.01 | -0.13, 0.11 |
| Positive | -0.06 | -0.19, 0.08 | 0.01 | -0.13, 0.16 |
| Smile | 0.16 | 0.00, 0.32 | 0.09 | -0.08, 0.25 |
| None of The Above | 0.00 | -0.31, 0.31 | -0.05 | -0.31, 0.21 |
| Caregiver facial expressions |  |  |  |  |
| Disgust | 0.25 | -0.25, 0.74 | -0.69 | -1.41, -0.01 |
| Mock Surprise | -0.16 | -0.30, -0.01 | -0.12 | -0.27, 0.02 |
| Negative | 0.26 | -0.02, 0.55 | -0.34 | -0.59, -0.09 |
| Neutral | 0.00 | -0.12, 0.12 | 0.01 | -0.12, 0.13 |
| Positive | -0.01 | -0.14, 0.11 | 0.00 | -0.13, 0.13 |
| Smile | -0.01 | -0.14, 0.12 | 0.02 | -0.11, 0.15 |
| Surprise | -0.15 | -3.01, 2.65 | -0.10 | -0.51, 0.30 |
| ‘Woe’ Face | -0.54 | -0.90, -0.16 | -0.51 | -0.87, -0.14 |
| None of The Above | -0.01 | -0.17, 0.16 | -0.15 | -0.33, 0.04 |

**Note.** This table presents the results from adjusted Model 5, examining associations between the Edinburgh Postnatal Depression Scale (EPDS) and the Standardised Assessment of Personality – Abbreviated Scale (SAPAS) scores and patterns of visual attention behaviours in infants and caregivers. The analyses were restricted to participants with complete data on both the EPDS and the SAPAS. Each row displays the adjusted regression coefficient (Beta) and the corresponding 95% Credible Interval (CrI) for the association between each specific visual attention behaviour and either the EPDS or SAPAS score. The EPDS is a 10-item self-report questionnaire assessing depressive symptoms in the postnatal period. The SAPAS is an 8-item interviewer-administered screening tool for personality disorder traits. CrI refers to the Bayesian 95% Credible Interval, representing the range within which the parameter is likely to fall with 95% probability. Positive coefficients indicate an association between higher EPDS or SAPAS scores and greater duration of the corresponding visual attention behaviour, while negative coefficients indicate an association with shorter duration. Credible intervals that do not include zero provide stronger evidence for an association. All models underlying the normalisation process were adjusted for the child’s age, child’s sex, parental age, caregiver identity (mother versus father), birth order of the child, and the type of head-mounted camera used (old versus new model).

STable 10. Frequencies, means, and standard deviations of child and parent normalised score of the proportion of total duration of visual attention by its modifiers (i.e., gaze direction).

|  | N_pduration0 (Infant) | N_pduration0 (Infant) |
| --- | --- | --- |
|  | **N, Mean (SD)** | **N, Mean (SD)** |
| Modifiers | Not possible to code gaze direction | Direct |
| Behaviours |  |  |
| Look at caregiver 1 | 42, 0.20 (0.84) | 63, 0.38 (0.90) |
| Look at caregiver 2 | 11, -0.70 (0.85) | 13, 0.28 (0.71) |
| Look at distraction | 19, 0.68 (0.54) | 20, 0.48 (0.66) |
| Look at focus object | 66, 0.27 (0.95) | 70, 0.27 (0.88) |
| Look at infant | - | - |
| Look at object outside of view | 59, 0.56 (0.82) | 54, 0.18 (0.96) |
| Look at other object | 19, 0.50 (0.63) | 35, 0.07 (1.28) |
| Look at other person | <5, 0.50 (0.33) | <5 |
| Look at sibling | 8, -0.24 (0.68) | 15, 0.21 (1.06) |
| No visual attention | - | - |
| Not possible to code visual attention | - | - |
|  | **N_pduration1 (Caregiver)** | **N_pduration1 (Caregiver)** |
|  | **N, Mean (SD)** | **N, Mean (SD)** |
| Modifiers | Not possible to code gaze direction | Direct |
| Behaviours |  |  |
| Look at caregiver 2 | 7, -0.36 (1.96) | 7, -0.73 (1.48) |
| Look at distraction | 20, -0.18 (1.20) | 24, 0.33 (1.18) |
| Look at focus object | 71, 0.04 (1.14) | 54, 0.33 (1.14) |
| Look at infant | 52, 0.35 (1.28) | 60, -0.50 (1.17) |
| Look at object outside of view | 53, 0.15 (1.18) | 56, 0.17 (1.03) |
| Look at other object | 39, 0.07 (1.15) | 42, 0.29 (1.09) |
| Look at other person | <5, 1.33 (-) | <5, -0.63 (1.20) |
| Look at sibling | 8, 0.54 (0.54) | 12, 0.61 (1.22) |
| No visual attention | - | - |
| Not possible to code visual attention | - | - |

**Note.** This table presents the frequencies, means, and standard deviations of the normalised score of the proportion of total duration of visual attention behaviours in infants and caregivers, categorised by gaze direction modifiers. The two modifiers included are "Not possible to code gaze direction" and "Direct gaze." The behaviours reflect different directions of visual attention, including looking at caregivers, distractions, objects, and other people. "N" refers to the number of observed behaviours, "Mean" indicates the mean normalised score of the proportion of total duration, and "SD" refers to the standard deviation. The normalised scores were derived from the total duration of visual attention behaviours relative to the overall recording time. Frequencies fewer than five observations are indicated as "<5" to protect participant anonymity. Missing values ("-") indicate that the behaviour was not observed in the corresponding gaze modifier category. All models underlying the normalisation process were adjusted for the child’s age, child’s sex, parental age, caregiver identity (mother versus father), birth order of the child, and the type of head-mounted camera used (old versus new model).

# **References**

Beebe, B. *et al.* (2010) ‘The origins of 12-month attachment: A microanalysis of 4-month mother–infant interaction’, *Attachment & human development*, 12(1–2), pp. 3–141.

Belotti, F. *et al.* (2015) ‘twopm: Two-part models’, *The Stata Journal*, 15(1), pp. 3–20.

Biringen, Z. *et al.* (2014) ‘Emotional availability (EA): Theoretical background, empirical research using the EA Scales, and clinical applications’, *Developmental Review*. Available at: https://doi.org/10.1016/j.dr.2014.01.002.

Bornstein, M.H. (no date) *A manual for coding mother-infant interaction.* Bethesda, MD USA.

Leckie, G. and Charlton, C. (2013) ‘Runmlwin: a program to run the MLwiN multilevel modeling software from within Stata’, *Journal of statistical software*, 52, pp. 1–40.

Lee, R. *et al.* (2017) ‘Through babies’ eyes: Practical and theoretical considerations of using wearable technology to measure parent–infant behaviour from the mothers’ and infants’ view points’, *Infant Behavior and Development*, 47, pp. 62–71. Available at: https://doi.org/10.1016/j.infbeh.2017.02.006.

Meins, E., & Fernyhough, C. (2015) ‘Mind-mindedness coding manual, Version 2.2.’, *Unpublished manuscript* [Preprint].

Skinner, A. *et al.* (2022) ‘Identifying stakeholder priorities in use of wearable cameras for researching parent-child interactions’. PsyArXiv. Available at: https://doi.org/10.31234/osf.io/9vxc7.

Spiegelhalter, D.J. *et al.* (2002) ‘Bayesian Measures of Model Complexity and Fit’, *Journal of the Royal Statistical Society Series B: Statistical Methodology*, 64(4), pp. 583–639. Available at: https://doi.org/10.1111/1467-9868.00353.
